# Targeting polo-like kinase 1, a regulator of p53, in the treatment of adrenocortical carcinoma

**DOI:** 10.1186/s40169-015-0080-3

**Published:** 2016-01-11

**Authors:** Kimberly J. Bussey, Aditi Bapat, Claire Linnehan, Melissa Wandoloski, Erica Dastrup, Erik Rogers, Paul Gonzales, Michael J. Demeure

**Affiliations:** NantOmics, LLC, The Biodesign Institute, Arizona State University, PO Box 875001, Tempe, AZ 85287-5001 USA; Translational Genomics Research Institute (TGen), Phoenix, AZ USA; Translational Drug Development (TD2), Scottsdale, AZ USA

**Keywords:** Adrenocortical carcinoma, PLK-1, p53, Targeted therapy

## Abstract

**Background:**

Adrenocortical carcinoma (ACC) is an aggressive cancer with a 5 year survival rate of 20–30 %. Various factors have been implicated in the pathogenesis of ACC including dysregulation of the G2/M transition and aberrant activity of p53 and MDM2. Polo-like kinase 1 (PLK-1) negatively modulates p53 functioning, promotes MDM2 activity through its phosphorylation, and is involved in the G2/M transition. Gene expression profiling of 44 ACC samples showed that increased expression of PLK-1 in 29 % of ACC. Consequently, we examined PLK-1’s role in the modulation of the p53 signaling pathway in adrenocortical cancer.

**Methods:**

We used siRNA knock down PLK-1 and pharmacological inhibition of PLK-1 and MDM2 ACC cell lines SW-13 and H295R. We examined viability, protein expression, p53 transactivation, and induction of apoptosis.

**Results:**

Knocking down expression of PLK-1 with siRNA or inhibition of PLK-1 by a small molecule inhibitor, BI-2536, resulted in a loss of viability of up to 70 % in the ACC cell lines H295R and SW-13. In xenograft models, BI-2536 demonstrated marked inhibition of growth of SW-13 with less inhibition of H295R. BI-2536 treatment resulted in a decrease in mutant p53 protein in SW-13 cells but had no effect on wild-type p53 protein levels in H295R cells. Additionally, inhibition of PLK-1 restored wild-type p53’s transactivation and apoptotic functions in H295R cells, while these functions of mutant p53 were restored only to a smaller extent. Furthermore, inhibition of MDM2 with nutlin-3 reduced the viability of both the ACC cells and also reactivated wild-type p53′s apoptotic function. Inhibition of PLK-1 sensitized the ACC cell lines to MDM2 inhibition and this dual inhibition resulted in an additive apoptotic response in H295R cells with wild-type p53.

**Conclusions:**

These preclinical studies suggest that targeting p53 through PLK-1 is an attractive chemotherapy strategy warranting further investigation in adrenocortical cancer.

**Electronic supplementary material:**

The online version of this article (doi:10.1186/s40169-015-0080-3) contains supplementary material, which is available to authorized users.

## Background

Adrenocortical carcinoma (ACC) is a rare and often fatal cancer. Recent studies have shown that the outcome for patients with ACC has remained essentially unchanged in the past 25 years, with the overall 5-year survival of patients undergoing surgical resection being approximately 40 % [1–3]. Patients who present clinically with large, locally invasive tumors resulting in involved surgical margins or those who present with metastatic disease fare considerably worse with 5 year survival rates of 10–20 % [1–3], largely due to the limited effectiveness of chemotherapy. The only realistic opportunity for cure is a complete surgical resection, but unfortunately metastatic spread is already present in 40-70 % of patients at the time of diagnosis precluding cure [2–8]. Standard chemotherapy remains based on mitotane (Bristol-Myers Squibb Company, New York, New York) which was first approved in 1960. Mitotane, also known as o,p’-DDD, is a derivative of the pesticide DDT and an adrenolytic [7, 9–11]. The response rates to mitotane as a single agent is a relatively poor 23 %, but survival for those patients whose tumors do respond is improved from 14 to 50 months [7, 9, 10, 12, 13]. For most patients, mitotane is poorly tolerated due to its severe toxic side effects, including the obliteration of the healthy contralateral adrenal gland. Although in the original phase II trial, etoposide, doxorubicin, and cisplatin in combination with mitotane (EDP-M), was reported to produce clinical response rates up to 49 % of patients in advanced ACC patients [14], the results were not substantiated in the phase III trial comparing this regimen to streptozocin and mitotane. In this trial, the response rate to EDP-M was 23.2 % and the median progression-free survival interval was 5 months [15]. Unfortunately, there is no approved second-line regimen for those whose disease progress on these agents.

Increasing, new treatments for cancer are being developed based on the analysis of genomic aberrations identified in tumor samples from patients. An improved understanding of the molecular oncogenesis of adrenocortical cancer (ACC) may elucidate novel therapeutic targets. The increased incidence of ACC in patients with Li-Fraumeni syndrome suggests the p53 pathway is involved in ACC progression [5, 6, 16]. In adults, however, mutation in p53 is seen in less than 25 % of cases suggesting that other elements of the p53 pathway may be perturbed [17–20]. Additionally, we and others have found that p53 is a major driver of differential gene expression when comparing ACC to normal adrenal glands or when comparing low and high-grade tumors [21, 22]. In the present analysis of gene expression profiles of ACC tumor samples, we found overexpression of polo-like kinase 1 (PLK-1), which is known to negatively regulate p53 activity, in 29 % of the tumors in our cohort and in 67 % of the tumors from the cohort published by Giordano, et al. [23]. It has also been shown to interact with the human murine double minute 2 (MDM2) and control its activity [24]. Therefore, we sought to delineate the role of PLK-1 in the modulation of the p53 pathway in ACC. We examined the effect of pharmacologic inhibition of PLK-1 on p53 function.

## Methods

### Gene expression data

We used previously published data sets from Affymetrix U133 Plus 2 chips that included both normal adrenal and ACC samples annotated with survival data [22, 23] to identify the number of ACC samples over-expressing PLK1. The.CEL files from Affymetrix U133 Plus 2 chips were downloaded from GEO (GSE10297 and GSE19750). The data were filtered to include only normal adrenal samples without evidence of disease in the contralateral gland and ACC tumor sample from patients over 18 years of age. Using the Expression File Creator in GenePattern [25], the data were normalized by gcRMA [26] with quantile normalization and background subtraction. Batch effects were minimized using COMBAT [27] with the non-parametric option, floored at 0.00001, and log2 transformed. Normalized expression data was extracted for the PLK1 probe set (202240_at), and the relative fold change to the geometric mean of the corresponding normal samples for each study was computed. The total data set encompassed 7 normal adrenal glands and 75 ACC. Of the ACC, 43 had usable survival information.

We validated the survival trend observed with array-based gene expression data using XENA [23] to pull out the mean normalized RSEM normalized count PLK1 expression for the gene and survival data from the TCGA ACC cohort. We used the built in Kaplan–Meier plotting function to determine the expression level cut-offs for a 2 group comparison for consistency, since there is no normal adrenal data in the TCGA data set.

To determine how SW-13 and H295R compared to tumors for PLK1 data, we mined GEO for PLK1 and beta-actin expression in SW-13 and H295 (the parental line of H295R) as well as all available ACC expression data sets. We downloaded the data and extracted the expression values for the corresponding probes to PLK1 and beta-actin, converted all data to log10, and plotted the results of PLK1 expression relative to beta-actin expression.

### Eukaryotic cell culture

SW-13 and H295R cell lines were both purchased from ATCC (Manassas, VA). SW-13 cells were cultured in DMEM media with 10 % Fetal Bovine Serum (FBS), 1 % Penicillin–Streptomycin and 1 % l-Glutamine (all from Gibco, Grand Island, NY). H295R cells were cultured in DMEM/F12 media with 1 % Penicillin–Streptomycin, 1 % l-Glutamine (all from Gibco, Grand Island, NY), 2.5 % NuSerum and 1 % insulin-transferrin-selenium (ITS) (both from BD Biosciences, San Jose, CA). All cell cultures were maintained in a 5 % CO_2_ atmosphere at 37 °C.

### In vitro drug dose–response curves

Sensitivity to BI-2536 (Tocris, Minneapolis, MN, USA), mitotane (Tocris, Minneapolis, MN, USA) and nutlin-3 (Tocris, Minneapolis, MN, USA) was tested in the following manner. H295R cells were plated at a density of 1750 cells/well in 40 µL of medium and SW-13 was plated at 1250 cells/wells in 40 µL DMEM with 2 % FBS. For dose response curves with BI-2536 alone, twenty-four hours after plating, threefold serial dilutions of BI-2536 in 10 μL of medium were added to the plates, with the final concentration at the highest dose being 100 µM. Cells were then incubated for an additional 72, 96, or 120 h at 37 °C in a humidified incubator. Viability was assessed by CellTiter Glo (Promega, Madison, WI, USA) and converted to normalized percent viability after normalizing to cells alone and with drug carrier. Double compound studies were conducted as above except IC_25_ concentrations (0.00684 µM for SW-13 and 0.0374 µM for H295R) of BI-2536 were kept constant and mitotane or nutlin-3 was evaluated in serial dilution. In those cases, normalized viability also included normalization to the median viability of BI-2536 alone.

Dose response curves and IC_50_ values for cell survival in the presence of the drugs were calculated using Prism5 software (GraphPad) using the log(inhibitor) vs. response—4 parameter function which fits the following equation: Y = Bottom + (Top–Bottom)/[1 + 10^(X-LogIC50)^] where X is the logarithm of concentration and Y is the percent cell survival. Y starts at the top and goes to bottom with a sigmoid shape as X increases. All experiments were repeated at least three times with a minimum of five replicates per dose per experiment and values are represented as averages with standard error.

### In vitro caspase 3/7 induction assays

Induction of apoptosis after treatment with BI-2536 and nutlin-3 alone or together in combination was determined using the Caspase 3/7 Glo assay (Promega, Madison, WI). H295R cells were plated at a density of 1750 cells/well in 40 µl of medium and SW-13 was plated at 1250 cells/wells in 40 µl DMEM with 2 % FBS. 24 h after plating, the cells were treated with 100 µM, 33.3 µM, 11.1 µM, 3.703 µM and 1.234 µM of BI-2536 and nutlin-3 in a 10 µl volume. Induction of apoptosis was determined by the cleavage of caspase 3/7 at 8, 16, 24 and 48 h after compound addition. 5 µM Doxorubicin (Tocris, Minneapolis, MN, USA) was used as a positive control for induction of apoptosis. Percent caspase 3/7 activity was normalized to cells and to DMSO control. Induction of apoptosis with double compounds was determined as above except IC_25_ concentrations (0.00684 µM for SW-13 and 0.0374 µM for H295R) of BI-2536 were kept constant and nutlin-3 was evaluated in serial dilution. In those cases, percent caspase 3/7 activity was also normalization to the median caspase 3/7 activity of BI-2536 alone.

### siRNA knockdown of PLK-1

Using 6 well plates, 2 × 10^5^ SW-13 and 3.5 × 10^5^ H295R cells were plated in their respective media without antibiotics and allowed to attach overnight. The next day, for SW-13 Lipofectamine2000 reagent (Invitrogen, Carlsbad, CA, USA) and for H295R TransIT-siQUEST reagent (Mirus Bio, Madison, WI, USA) was used to transfect in 20 nM PLK-1 siRNA (Qiagen, Valencia, CA, USA), an all-stars negative siRNA (Qiagen, Valencia, CA, USA) as a negative control, or a universally lethal positive-control siRNA directed against ubiquitin B (UBBs1) [28] (Qiagen, Valencia, CA, USA) using the manufacturer’s recommended protocols respectively. For transfection of SW-13 cells, 5 µl of Lipofectamine 2000 and 20 nM PLK-1, negative-control, or UBBs1 siRNA were mixed together in equal volumes, in serum free media (SFM) and allowed to incubate at room temperature for 30 min. All the media from the SW-13 wells was aspirated off and 500 µl of the siRNA—Lipofectamine 2000 mix was added to each well along with 1.5 ml of media without antibiotics. For transfection of H295R cells, 4 µl of TransIT-siQUEST reagent was diluted in SFM. 20 nM PLK-1, negative-control or UBBs1 siRNA were added to the diluted transfection reagent and allowed to incubate at room temperature for 20 min. 250 µl of the siRNA—TransIT-siQUEST mix was added to each well containing 1.25 ml of media without antibiotics. PLK-1 protein knockdown was confirmed by immunoblot 72 h after transfection.

To determine cell viability after PLK-1 transfection, SW-13 cells were reverse transfected with siRNA to PLK-1 or control siRNA and assayed for viability after 96 h. Briefly, 384 well plates were printed with 20 nM of Hs_PLK-1_7 siRNA, UBBs1, or negative control siRNAs that included a non-silencing scrambled siRNA and a siRNA directed against green fluorescent protein (GFP). A total of 20 µl of diluted Lipofectamine2000 solution was added to each well. After 30 min, 1200 cells for SW-13 in 20 µl of medium were added per well and then cultured at 37 °C. After 96 h, viability was assessed by CellTiter Glo following the manufacturer’s protocol. Relative luminescence values were normalized to cells and to cells with transfection agent to get normalized percent viability. To determine viability of H295R cells after PLK-1 transfection, H295R were transfected with PLK-1, negative-control or UBBs1 siRNA and assayed for viability after 72 h. Briefly, 20,000 H295R cells were plated in 80 µl in 96 well plates and allowed to attach overnight. The next day 20 µl of TransIT-siQUEST reagent and 20 nM PLK-1, negative-control or UBBs1 siRNA in SFM were added to each well containing 80 µl of media without antibiotics. Cells were assayed for viability using CellTitre Glo 72 h after transfection as per the manufacturer’s protocol. Relative luminescence values were normalized to cells alone and then cells with transfection agent alone to get normalized percent viability.

### Total RNA extraction

6 × 10^5^ SW-13 and 7.5 × 10^5^ H295R cells were plated in 10 cm^2^ dishes in 10 ml of media. Cells were allowed to adhere for 24 h and were then treated with their respective BI-2536 IC_10_, IC_25_ and IC_50_ doses including DMSO controls. Total RNA was extracted 24 h later using the mirVana miRNA Isolation Kit (Ambion, Inc, Grand Island, NY, USA) as per the manufacturer’s protocol for total RNA extraction. This kit efficiently extracts RNAs larger than 10 nucleotides with specific protocols for either total RNA extraction or small RNA extraction. This allows us to use the kit for all experiments, reducing variability due to differences between kits.

### RT-qPCR validation of p53 and p21 mRNA levels

Total RNA was reverse transcribed utilizing both random hexamer and oligo-dT primers and the RT^2^ First Strand cDNA Synthesis Kit (SABiosciences, Valencia, CA, USA). The resulting cDNA was amplified on the iQ5 Real-Time PCR Detection System (Bio-Rad Laboratories, Inc, Hercules, CA, USA) using primer sets for TP53 (p53), CDKN1A (p21) and ACTB (β-actin) and RT^2^ SYBR Green Master Mix (all from SABiosciences, Valencia, CA, USA) and run according to manufacturer’s instructions. Melting curve analysis was performed to evaluate primer set specificity. Fold difference with respect to the vehicle control, DMSO, and relative to β-actin, which was used as the reference gene, was calculated using the Pfaffl method taking into account reaction efficiencies [29].

### Western blot analysis

6 × 10^5^ SW-13 and 7.5 × 10^5^ H295R cells were plated in 10 cm^2^ dishes in 10 ml of media. Cells were allowed to attach for 24 h after which they were treated with IC_10_, IC_25_, IC_50_ concentrations of BI-2536 (Table 1) and DMSO vehicle control for 24 h. Cells were lysed with RIPA buffer with protease and phosphatase inhibitors, and the resulting protein lysate quantitated by BCA (Pierce, Thermo Scientific, Pittsburgh, PA, USA). Thirty micrograms of protein were loaded onto 4–12 % Bis–Tris pre-cast gels (Invitrogen, Carlsbad, CA, USA) and allowed to separate at 150 V for 1 h. The gels were then transferred onto PVDF membranes for 7 min using the iBLOT western transfer system (Invitrogen, Carlsbad, CA, USA) at room temperature. Following the transfer of proteins, the membranes were blocked in 5 % blocking solution made from, non-fat dry milk dissolved in 1x TBST (50 mM Tris.HCl, pH 7.4, 150 mM NaCl + 0.1 % Tween 20) for 1 h. Primary PLK-1 antibody (Cell Signaling, Technology, Danvers, MA, USA) at a dilution of 1:500 was added to membranes in 5 % BSA (1× TBST + 5 % BSA) overnight at 4 °C. Primary antibodies to MDM2 (AbCam, Cambridge, MA, USA) and p53 (AbCam, Cambridge, MA) at a dilution of 1:500 were added to the membranes in 1 % blocking solution overnight at 4 °C. The next day the membranes were washed with 1× TBST twice for 10 min and appropriate HRP labeled secondary anti-rabbit (Cell Signaling Technology, Danvers, MA, USA) for PLK-1 and anti-mouse (Cell Signaling Technology, Danvers, MA, USA) for MDM2 and p53 antibodies were added to the blots at a dilution of 1:1000 for 2 h at room temperature. The blots were then washed in 1x TBST four times for 10 min. The membranes were developed using the SuperSignal West Femto Chemiluminescent Substrate (Pierce, Thermo Scientific, Pittsburgh, PA, USA) and were visualized and quantitated with the Bio Spectrum 500 Imaging System (UVP, Cambridge, UK).

**Table 1:** BI-2536 inhibitory concentrations

Cell line	BI-2536 (µM)		
	IC_10_	IC_25_	IC_50_
H295R	0.00565	0.00684	0.0095
SW-13	0.0222	0.0374	0.0628

The blots were processed as described above for the detection of β-Actin (AbCam, Cambridge, MA, USA) in 5 % blocking solution which was used as an internal loading control. The β-Actin antibody was used at a dilution of 1:1000 along with the anti-rabbit secondary antibody also at a dilution of 1:1000. The actin membranes were also detected using the SuperSignal West Femto Chemiluminescent Substrate (Pierce, Thermo Scientific, Pittsburgh, PA, USA) and were visualized and quantitated with the Bio Spectrum 500 Imaging System (UVP, Cambridge, UK) and relative amounts of PLK-1, MDM2 and p53 protein are reported relative to β-Actin. All experiments were done three individual times and are represented as averages with standard error.

To determine PLK-1 protein expression after siRNA knockdown, blots for PLK-1 and β-Actin were processed as described above. Relative amounts of the PLK-1 protein after siRNA knockdown are reported relative to β-Actin. All experiments were done three individual times and are represented as means with standard error.

### In vivo sensitivity assays

For in vivo testing of drug efficacy, xenograft models in female ncr nude mice were used. H295R and SW-13 were utilized in the creation of mouse xenograft tumors. All procedures were carried out under the institutional guidelines of TGen Drug Development’s Institutional Animal Care and Use Committee (Protocol #06001, initially approved January 2006). To create xenografts, 5.0 × 10^6^ cells were injected subcutaneously in a solution of 50 % media/50 % Matrigel™ into the right flank of the animal. Cohorts were assigned by random-equilibration into groups of 10 for SW-13 or groups of 9 for H295R. On Day 1, mitotane was diluted with a corn oil solution immediately prior to dosage administration to working concentration of 30 mg/ml in order to deliver 300 mg/kg doses at a 0.2 ml fixed dose volume. Mitotane was given daily by oral gavage administration for 20 days. BI-2536 (30 mg/kg) was administered twice weekly by IP injection [30]. When the mean tumor weight of the control group reached 1500 mg, the study was terminated. Tumors were measured using Vernier calipers and tumor weight calculated using the formula: Tumor weight (mg) = (a × b2/2) where ‘b’ is the smallest diameter and ‘a’ is the largest diameter. Body weights were recorded when the mice were pair-matched. In addition, body weights were taken twice weekly thereafter in conjunction with tumor measurements. Mean tumor growth inhibition (TGI) was calculated utilizing the following formula:

$${\text{TGI}} = \left[ {1 - \frac{{\bar{\chi }_{{{\text{Treated}}\; ( {\text{Final)}}}} - \bar{\chi }_{{{\text{Treated}}\, ( {\text{Day1}})}} }}{{\bar{\chi }_{{{\text{Control}}\,\; ( {\text{Final)}}}} - \bar{\chi }_{{{\text{Control}}\, ( {\text{Day1)}}}} }} \times 100\,\% } \right]$$

Tumors that regressed from the Day 1 starting size were removed from the calculations. All statistical analyses in the xenograft study were performed with GraphPad Prism^®^ v4 software. Differences in final tumor weights were confirmed using the Analysis of Variance (ANOVA) with Tukey’s Multiple Comparison Test.

### Statistical analysis

All statistical analyses were done with the Prism6 software (GraphPad) and included Analysis of Variance (ANOVA) with Tukey’s Multiple Comparison Test, Two-Way ANOVA with Dunnett’s multiple comparison test, and Survival using the log-rank method as implemented by the software. Cox Proportional Hazards were computed in R using the Survival package with stratification of samples by residual tumor status. P-values below 0.05 were considered strong a strong indication of non-random results, but where we report a trend, a *p* value is also reported even if it was above 0.05 to allow the reader to judge the non-random nature of the trend.

### Evaluation of p53 and MDM2 genotypes

Studies of p53 mutations, p53 codon 72 polymorphism, and MDM2 SNP309 polymorphism are detailed in the Additional file 1, as the results of the studies were largely negative.

## Results

### Inhibition of Polo-like kinase 1 (PLK-1) reduced the viability of ACC cell lines

**Fig. 1:**
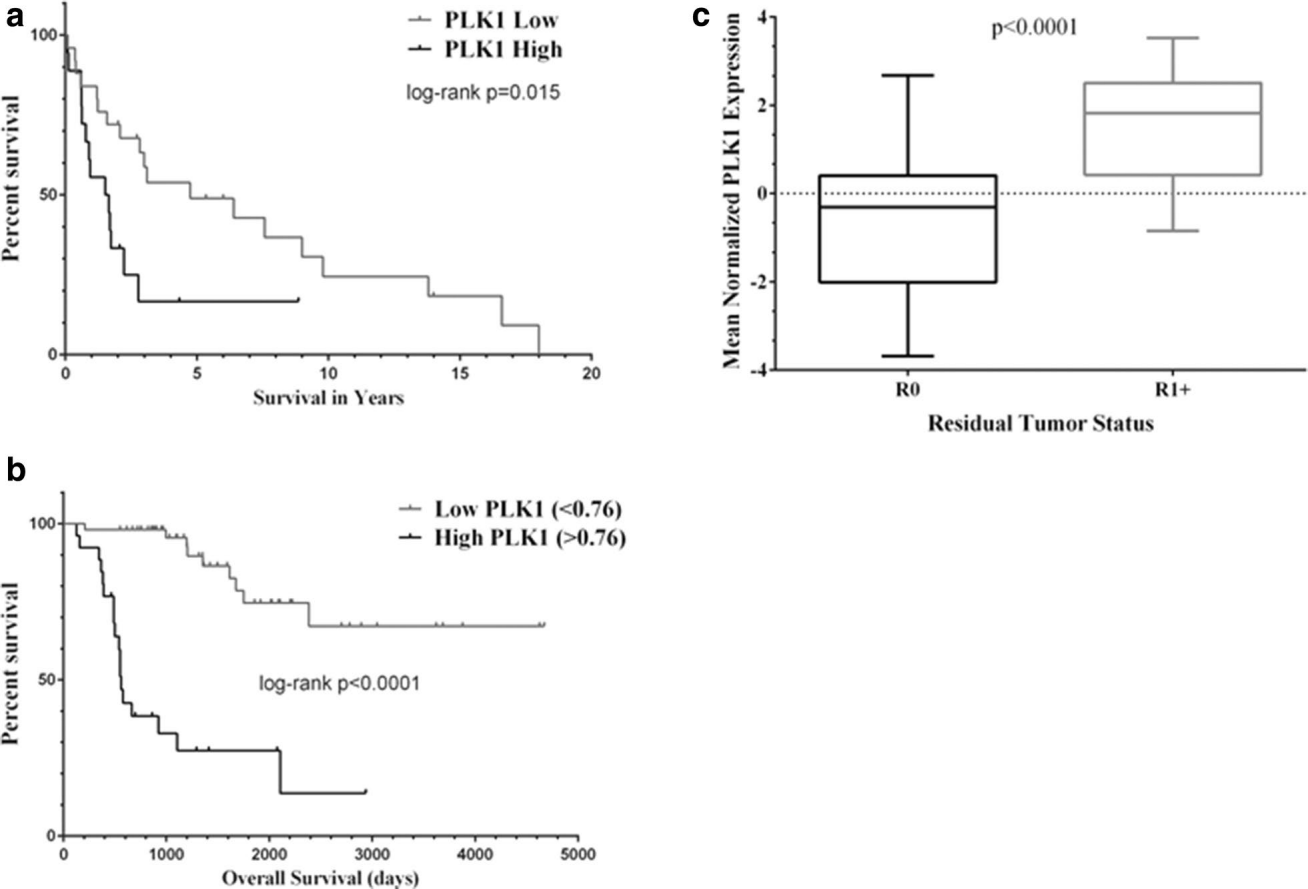
High PLK1 mRNA expression is associated with worse prognosis in ACC tumors. **a** In data from Affymetrix U133 plus 2 arrays, tumors that exhibit a log2 fold-change of 2 or greater relative to normal have a worse overall survival compared to tumors that express less PLK1 mRNA. **b** The relationship between PLK1 mRNA expression and shorter survival times was validated by RNA sequencing data from TCGA. **c** In the TCGA data set, the only clinical parameter associated with PLK1 expression is residual tumor status

Examination of our gene expression data [22] combined with that of Giordano, et al. [23]. revealed that PLK1 was over-expressed in 37 % of tumors with a mean log2 fold-change relative to normal adrenal of 7.88 (range −0.0589 to 30.74, Additional file 1: Table S3). There was no correlation between the expression levels of PLK1, MDM2, and TP53. Since increased PLK-1 expression has been correlated to poor prognosis and aggressiveness of cancers, we evaluated whether a similar association exists in ACC. We found that patients with tumors that had 2 fold or greater over-expression had a median survival of 1.585 years compared to a median survival 4.753 years for patients with tumors having near normal PLK1 expression levels (Fig. 1a), yielding a log-rank hazard-ratio (HR) of 2.188 (95 % confidence interval 1.289–6.207). We obtained similar results using the gene average PLK1 expression data from TCGA for ACC (Fig. 1b), with a log-rank HR of 7.773 (95 % confidence interval 7.441–48.28). We used the TCGA data to examine clinical correlates. The only correlation observed was the residual tumor status. Tumors classified as R0 had less expression of PLK1 compared to tumors that were classified as R1, R2 or RX (Fig. 1c). The Cox proportional HR for PLK1 expression taking into account the residual tumor status was 1.626 (95 % confidence interval 1.124–2.351, p = 0.00979).

**Fig. 2:**
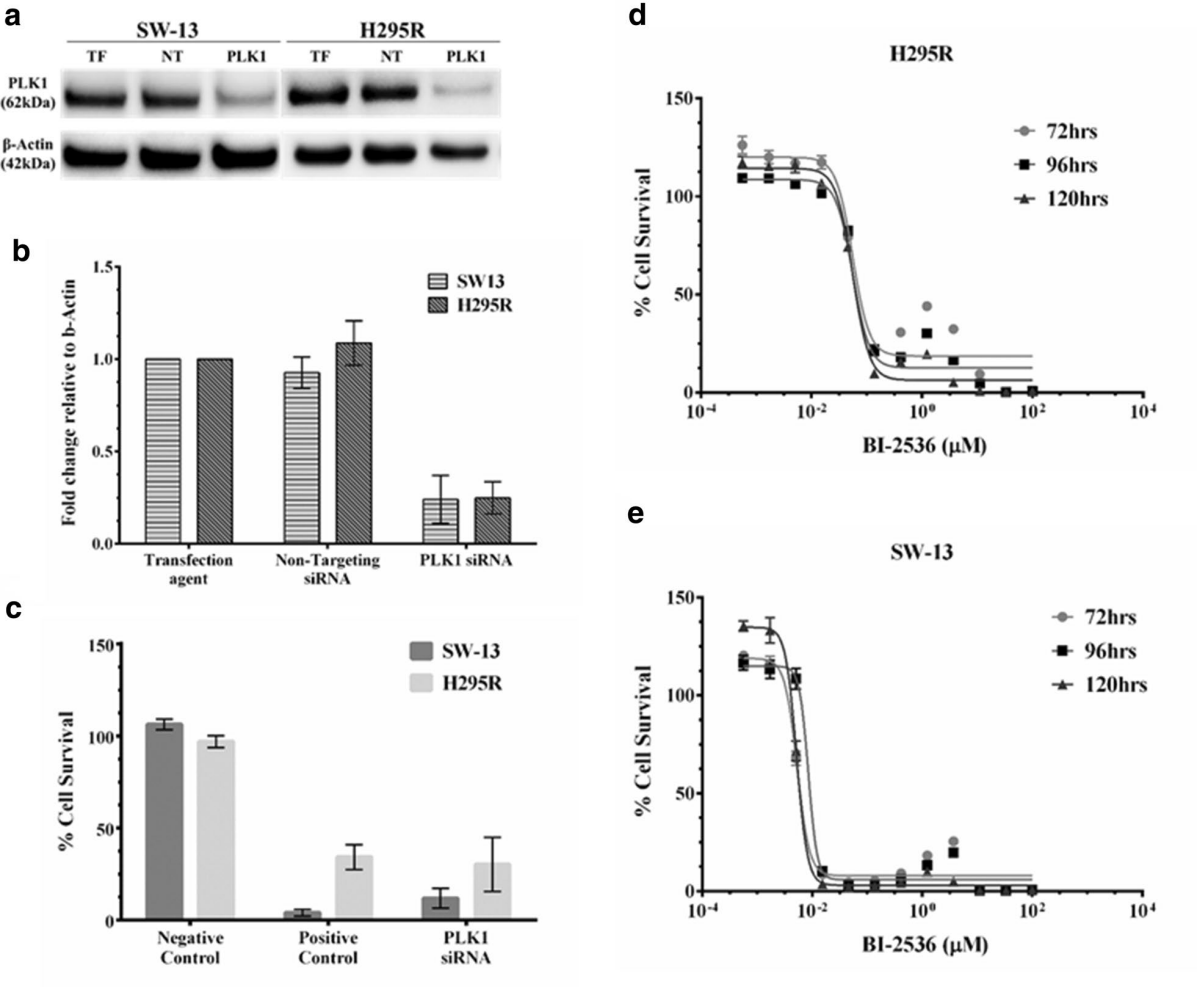
Inhibition of Polo-like kinase 1 (PLK-1) reduced the viability of ACC cell lines: Given that PLK-1 is a negative regulator of p53, inhibition of PLK-1 should reduce viability of the ACC cell lines. **a** ACC cell lines, H295R and SW-13 were treated with PLK-1 siRNA and amount of PLK-1 protein was determined after 72 h of siRNA treatment. **b** Quantitation of PLK-1 protein western after siRNA knockdown expressed relative to β-actin. **c** Knocking down expression of the PLK-1 protein reduced viability of the H295R & SW-13 cell lines as compared to the controls. **d**, **e** Similarly, treating the cells with BI-2536, an inhibitor of PLK-1, reduced the viability of both the H295R and SW-13 ACC cell lines. Assays were normalized first to cells alone and then to a vehicle control. This resulted in viability measures of greater than 100 %. All experiments were done at least three individual times and data are represented as means with standard error. *TF* Transfection agent and *NT* Non-targeting siRNA. Uncropped images of full blots with molecular weight markers can be found in the Additional file 1: Figure S1

Next we wanted to determine the effect of downregulating PLK-1 on the ACC cell lines. We found that knocking down expression of PLK-1 using siRNA not only reduced the amount of PLK-1 protein (Fig. 2a, b), but also decreased the viability of both H295R and SW-13 to levels close to that of the positive control, UBB1, after siRNA knockdown (Fig. 2c), demonstrating that the ACC cell lines were very sensitive to the loss of PLK-1.

Similarly, exposure to the PLK-1 inhibitor BI-2536 showed that both H295R and SW-13 cell lines were also extremely sensitive to PLK-1 inhibition (Fig. 2d, e). H295R had an IC_50_ value of 0.063 µM while SW-13 was ~tenfold more sensitive compared to H295R with an IC_50_ value of 0.0095 µM 120 and 96 h following addition of drug respectively (Fig. 2d, e; Table 2). These values are well below the concentrations achieved clinically (31.2–55.2 µM) following a single intravenous injection of 50–70 mg of BI-2536 [31], but well with in the range of IC_50_ responses seen in a variety of cancer cell lines (Additional file 1: Figure S2).

**Table 2:** IC_50_ values of BI-2536 and nutlin-3 alone and in combination

IC_50_ values	H295R	SW13
BI-2536	0.0628 µM	0.0094 µM
Nutlin-3	12.75 µM	19.78 µM
Nutlin-3 + BI-2536	2.838 µM	6.502 µM
Combination Index	0.22	0.33

**Fig. 3:**
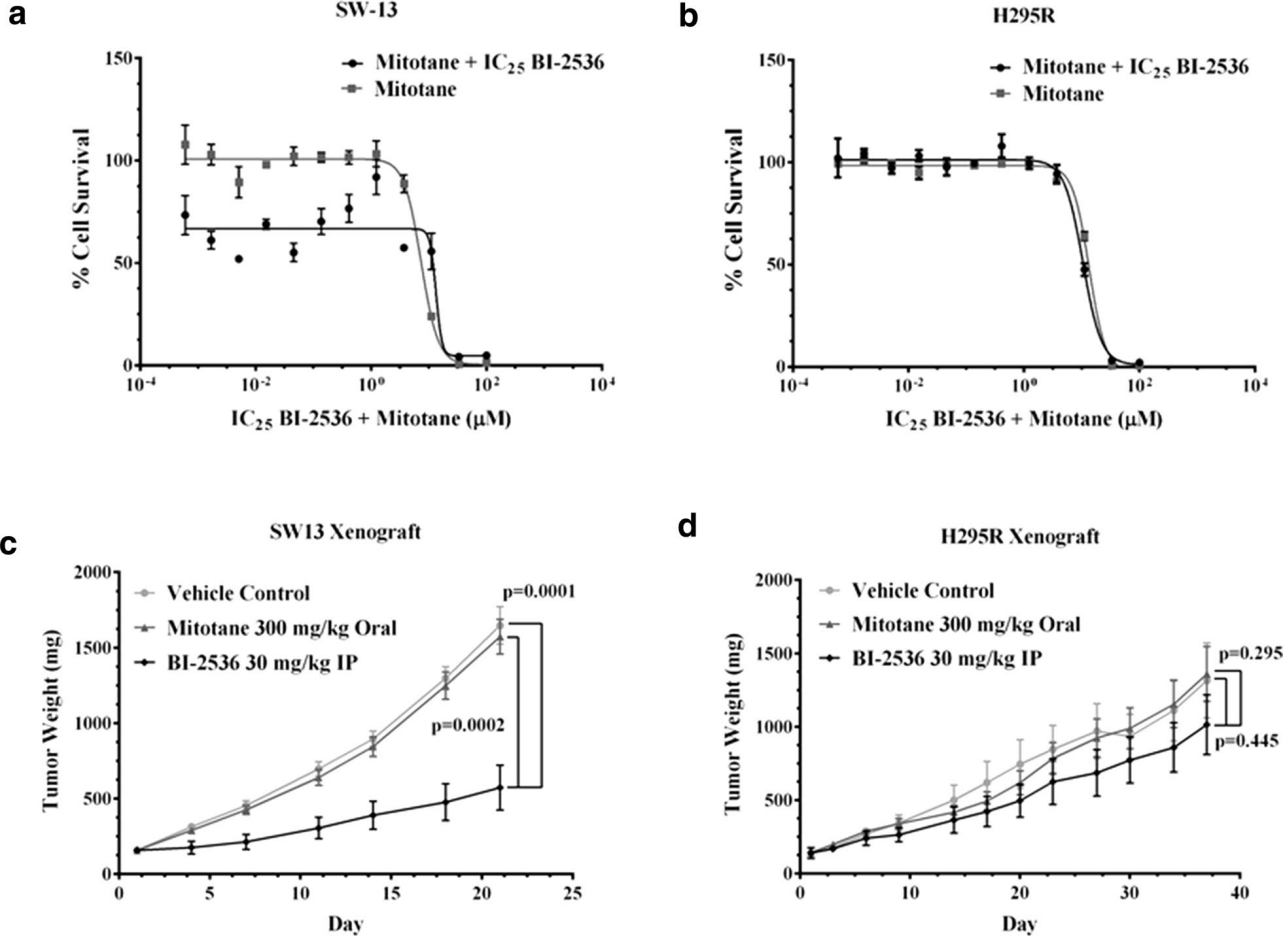
Effect of BI-2536 and mitotane on SW-13 and H295R cell lines and their tumor xenografts: Mitotane and BI-2536 did not lead to increased cell death in either SW-13 (**a**) or H295R (**b**) as compared to mitotane alone. The above experiments were done 3 individual times and data are presented as means with standard error. BI-2536 showed inhibited tumor growth to a greater extent in xenografts of SW-13 (p = 0.0001 versus vehicle, p = 0.0002 versus mitotane, **c** compared to the H295R xenograft model (p = 0.445 versus control, p = 0.0295 versus mitotane, (**d**). The difference in response may be due to the difference in p53 status of the cell lines. SW-13 is heterozygous for a mutation in p53 while H295R is wild-type for p53

As mitotane is the current standard of care for patients with advanced ACC [2, 4, 11, 13], we wanted to determine whether inhibition of PLK-1 could sensitize ACC cell lines to mitotane. Combined treatment with BI-2536 and mitotane did not increase the sensitivity of SW-13 or H295R ACC cell lines to mitotane (Fig. 3a, b).

We also studied the efficacy of BI-2536 in a murine xenograft model of ACC. Marked inhibition of tumor growth in response to BI-2536 relative to either vehicle control or mitotane-treated tumors was observed for SW-13 xenografts while the inhibition in H295R (Fig. 3c, d) was less dramatic, recapitulating the in vitro cell viability data (Fig. 3a, b; Table 3). The lack of response of the xenografts to mitotane is not surprising given that clinically less than 25 % of patients respond to the drug alone or in combination [11, 12, 15]. Thus, PLK-1 loss either by silencing with siRNA or through pharmacological inhibition with BI-1536 dramatically impacts ACC cell viability both in vitro and in vivo.

**Table 3:** IC_50_ values mitotane alone and in combination with BI-2536

IC_50_ values	H295R	SW13
Mitotane	13.45 µM	7.36 µM
Mitotane + BI-2536	10.49 µM	13.34 µM
Combination Index	0.78	1.81

### Inhibition of PLK-1 reduced levels of mutant p53 protein but not wild type p53

**Fig. 4:**
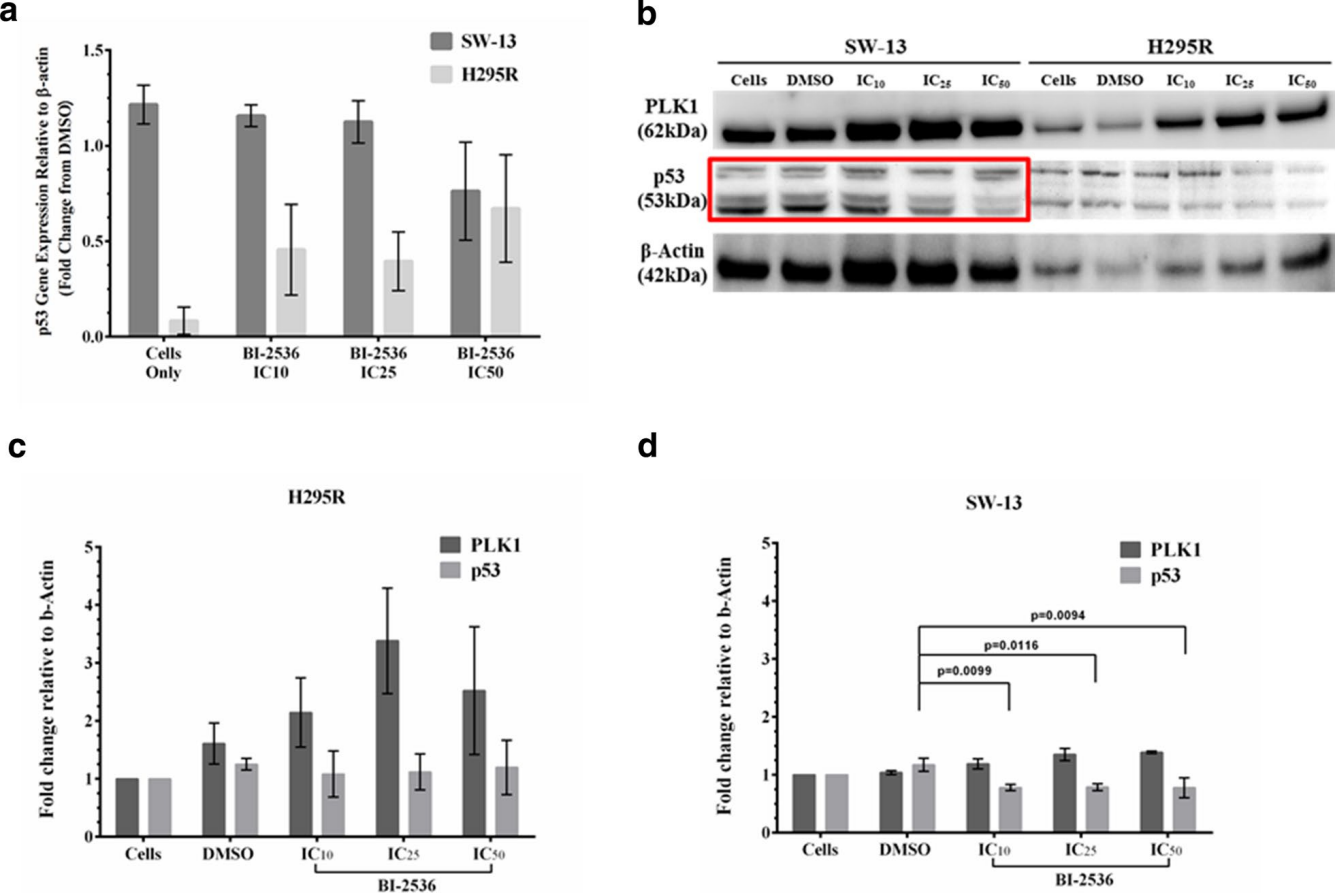
Inhibition of PLK-1 reduces expression of mutant p53 protein in SW13 cells: Since PLK-1 has been shown to physically interact with p53 to control its functions; the levels of the p53 protein should not be affected after inhibition of PLK-1. **a** Inhibition of PLK-1 with BI-2536 did not reduce transcript levels of either wild type or mutant p53 as determined by qRT-PCR. **b** However, BI-2536 treatment resulted in a decrease in the amount of mutant p53 protein in the SW-13 cells, but not of the wild type p53 protein in the H295R cells. **c**, **d** Quantitation of PLK-1 and p53 protein westerns after BI-2536 treatment expressed relative to β-actin. This confirms that p53 protein expression decreased in SW-13 and remains constant in H295R. All experiments were done at least 3 individual times and data are represented as means with standard error. The same western blots were stripped and re-probed for multiple proteins. Thus, the blots were cropped for ease of presentation. Uncropped blots with molecular weight standards can be found in the Additional file 1: Figure S2

PLK-1 is a negative modulator of p53 activity but has not been shown to affect its levels [32]. Therefore, we would predict that inhibition of PLK-1 would not affect p53 protein levels in ACC. Treatment with BI-2536 did not affect the levels of p53 transcript in either of the two cell lines (Fig. 4a). However, using an antibody that recognizes both mutant and wild-type p53 with its multiple isoforms, we saw a dose-dependent decrease of mutant p53 protein levels in SW-13 cells but not in the wild-type p53 protein levels in H295R cells (blots—Fig. 4b; quantification of blots Fig. 4c, d).

### Inhibition of PLK-1 restores functioning of wild type p53

**Fig. 5:**
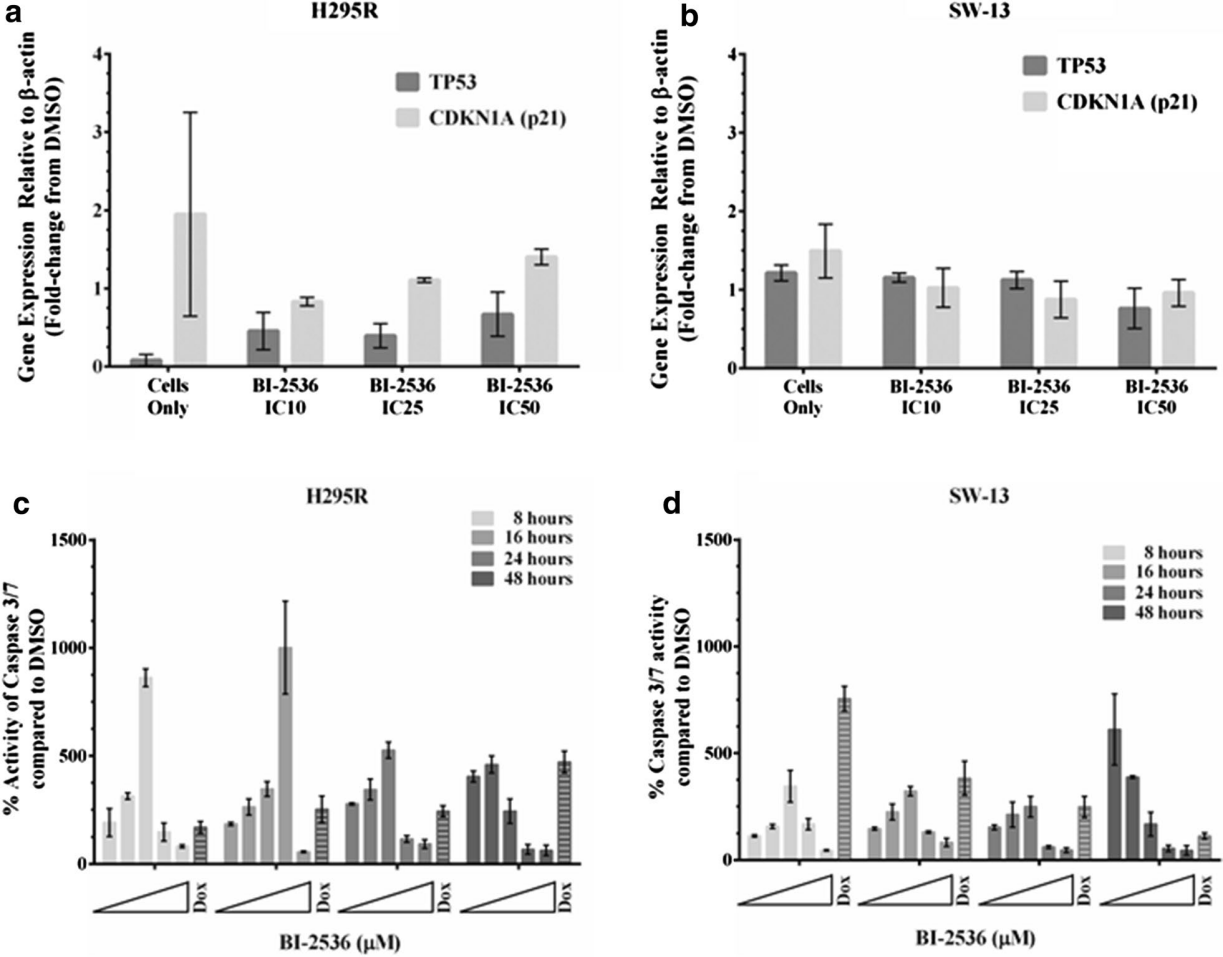
Inhibition of PLK-1 restores p53 functioning: Since PLK-1 is a negative regulator of p53, inhibition of PLK-1 should restore p53 transactivation and apoptotic functions. **a** Treatment of H295R and SW-13 cells with BI-2536 resulted in the restoration of wild type p53’s transactivation functions as seen by the increased transcription of its downstream gene, CDKN1A (p21) determined by qRT-PCR. **b** However, BI-2536 treatment of the SW-13 did not increase the transcription of CDKN1A, as it is mutant for p53 and the mutation is predicted to decrease p53 transactivation functions. **c** Furthermore, inhibition of PLK-1 restored wild type p53’s apoptotic response in H295R cells as determined by the Caspase 3/7 Glo assay. **d** The apoptotic response was slightly delayed in the SW-13 cells with mutant p53 with maximum response observed at 48 h. Doxorubicin was used as a positive control for apoptosis. All experiments were done at least 3 individual times, and data are represented as means with standard error

Since PLK-1 is a negative modulator of p53, inhibition of PLK-1 would relieve the negative regulation on wild-type p53 and potentially restore its transactivation and apoptotic functions. In the context of the predicted non-functional transactivation of the mutant p53 in SW-13, we would expect little impact on transactivation and unknown impact on apoptosis, particularly in light of the loss of mutant p53 protein upon BI-2536 exposure. We assessed p53’s transactivation and apoptotic functions after treatment with BI-2536. Inhibition of PLK-1 with BI-2536 resulted in a slight increase in the transcription of CDKN1A (gene that encodes the p21 protein) in H292R cells with wild type p53 (Fig. 5a). On the other hand, a decrease in mutant p53 protein in SW-13 cells after exposure to BI-2536 also reduced the amount of CDNK1A message (Fig. 5b). Furthermore, we tested whether inhibition of PLK-1 with BI-2536 could also restore p53’s apoptotic function. Treatment with BI-2536 resulted in a robust induction of apoptosis after treatment with BI-2536 in H295R cells (Fig. 5c) and to a lesser extent in SW-13 cells with mutant p53 (Fig. 5d). However, maximum induction of apoptosis in SW-13 cells was observed at 48 h, while only high concentrations of BI-2536 resulted in an increase in apoptosis at early time points (Fig. 5c, d).

### Synergy of PLK-1 inhibition by BI-2536 with MDM2 inhibition by nutlin-3

**Fig. 6:**
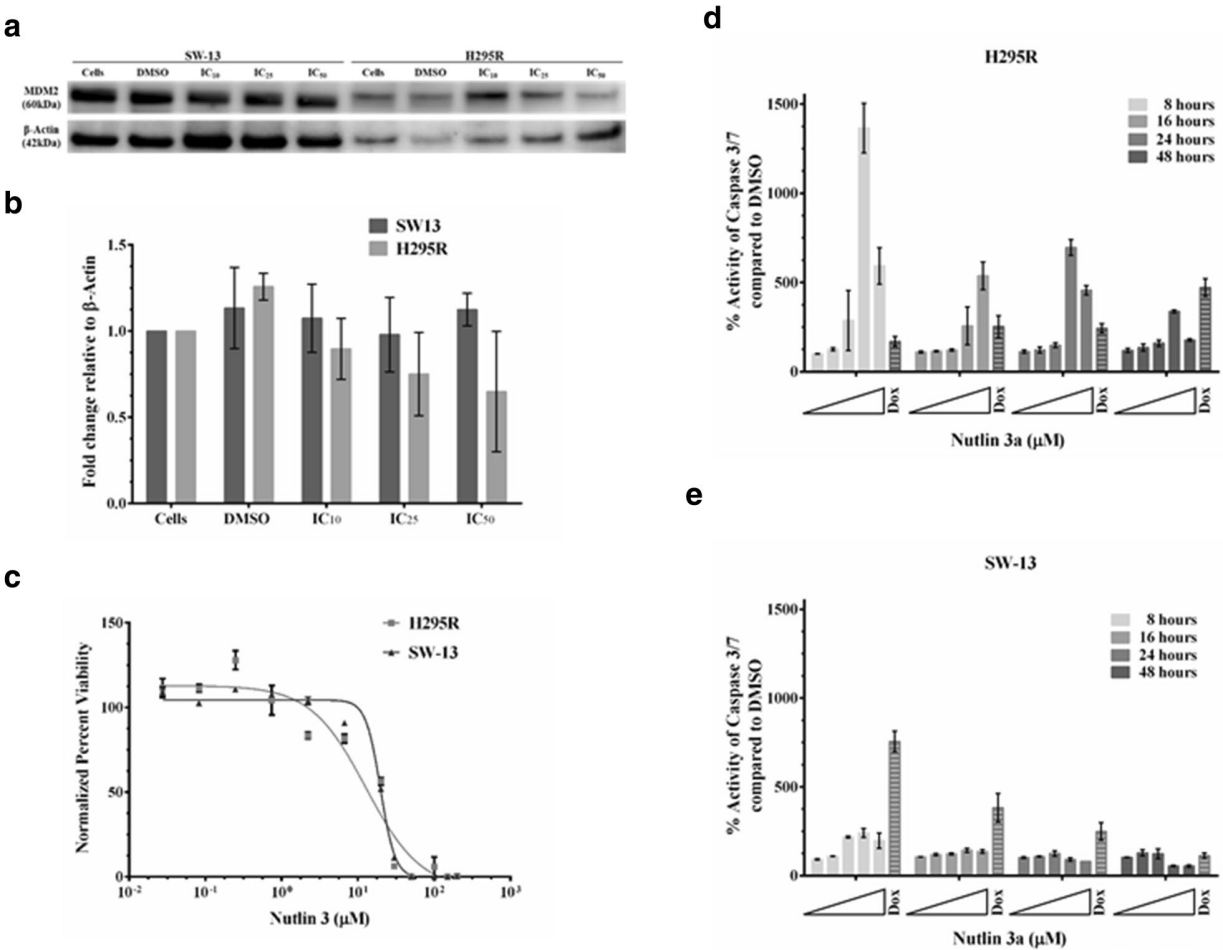
Inhibition of MDM2 by nutlin-3 restored wild type p53’s apoptotic response: Since PLK-1 also indirectly controls p53’s activity via MDM2, we determined the effect of PLK-1 inhibition on MDM2 functioning. **a** Inhibition of PLK-1 with BI-2536 did not change the levels of the MDM2 protein. **b** Quantitation of the MDM2 protein western after BI-2536 treatment expressed relative to β-actin. **c** Treating the cells with nutlin-3, an inhibitor of MDM2, reduced the viability of both the H295R and SW-13 ACC cell lines. **d** Furthermore, inhibition of MDM2 restored wild type p53’s apoptotic response in H295R cells as determined by the Caspase 3/7 glo assay. **e** However, a very small increase in apoptosis was observed in the SW-13 cells with mutant p53. Doxorubicin was used as a positive control for apoptosis. All experiments were done at least three individual times and data are represented as means with standard error. Uncropped blots that include molecular weight standards can be found in the Additional file 1: Figure S3

**Fig. 7:**
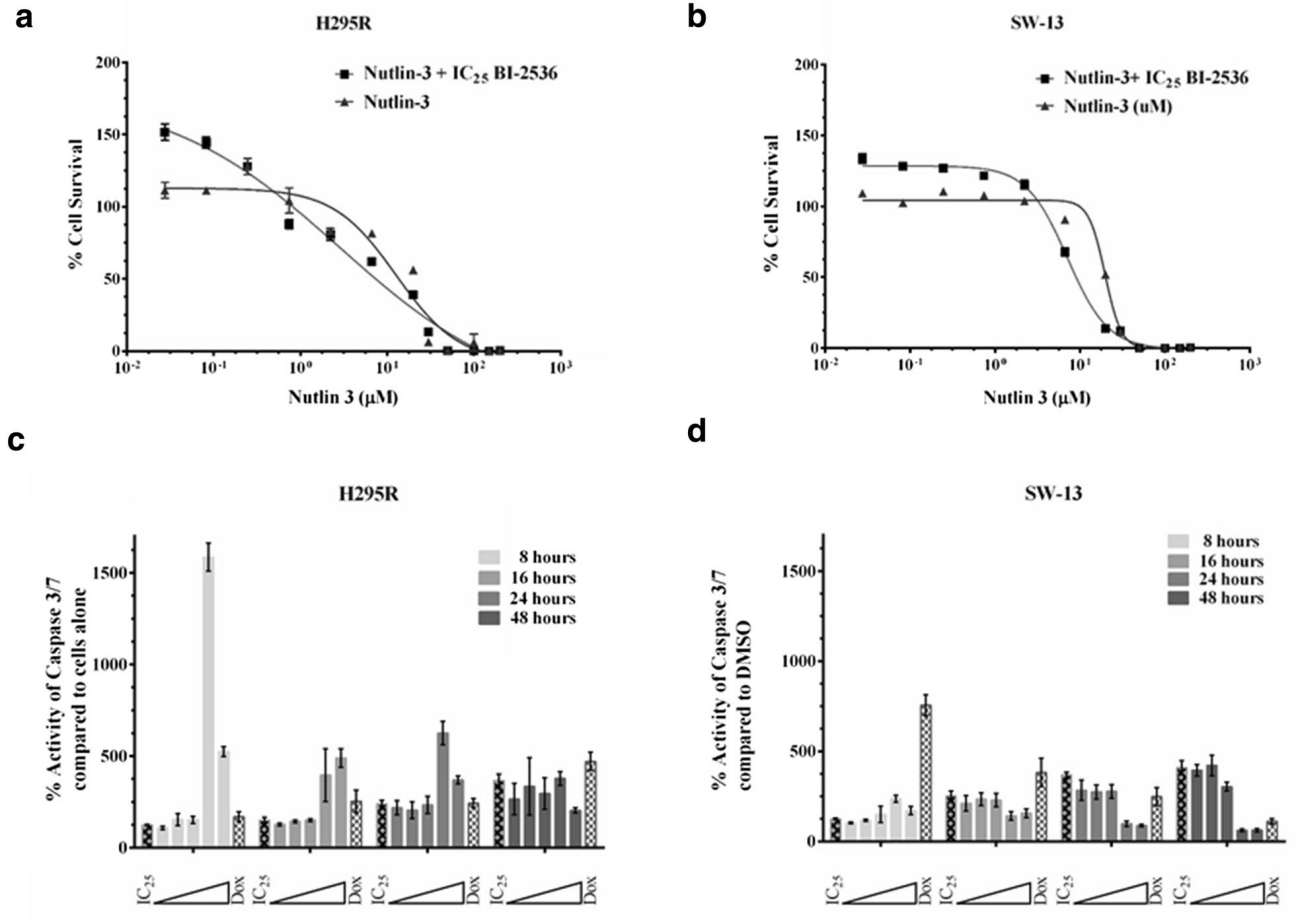
Synergy of PLK-1 inhibition by BI-2536 with MDM2 inhibition by nutlin-3: Since PLK-1 plays a role in controlling p53 directly and via MDM2, we wanted to determine whether inhibition of PLK-1 would show synergy with MDM2 inhibition. **a**, **b** Indeed treatment of H295R (**a**) and SW-13 (**b)** cells with nutlin-3 and BI-2536 sensitized both the cell lines to nutlin-3. We also anticipated that dual inhibition of PLK-1 and MDM2 would result in a synergistic apoptotic response via p53. **c**, **d** Dual inhibition of PLK-1 and MDM2 resulted in an additive response in the H295R cells (**c**), with wild-type p53, whereas no additive apoptotic response was seen in the SW-13 cells (**d**) with mutant p53. Doxorubicin was used as a positive control for apoptosis. All experiments were done at least three individual times and data are represented as means with standard error

While both PLK-1 and MDM2 regulate p53 activity independently, they also cooperate to modify p53 function. PLK-1 phosphorylates MDM2 at serine 260, stimulating the activity of MDM2 and increasing the turnover of p53 [24]. We therefore also determined the effect of BI-2536 treatment on MDM2 protein levels and found that inhibition of PLK-1 did not affect the levels of the MDM2 protein (Fig. 6a, b). We also determined whether inhibiting MDM2 with nutlin-3, an inhibitor of MDM2 would reduce viability of ACC cells, presumably as a result of the restoration of p53 functions. Treating both H295R and SW-13 cells with nutlin-3 decreased their cell viability (Fig. 6c) and restored wild type p53’s apoptotic response in H295R cells (Fig. 6d, e). Given that PLK-1 directly modulates p53 functions as well as indirectly by modulating MDM2 functions, we wanted to determine inhibiting PLK-1 would sensitize cells to the effects of nutlin-3, an inhibitor of MDM2. We observed a decrease in the IC_50_ values of nutlin-3 when it was combined with the IC_25_ concentration of BI-2536 after controlling for the effect of BI-2536 itself in both the cell lines (Fig. 7a, b; Table 2). This effect was independent of p53 mutation status, as both ACC cell lines responded in a similar fashion.

Inhibition of PLK-1 and MDM2 should result in the reactivation of p53’s apoptotic function, due to dual relief of p53 inhibition [33]. However, combined inhibition of PLK-1 and MDM2 did not increase apoptotic response of SW-13 over that seen with BI-2536 alone, whereas an additive apoptotic response was observed in H295R cells with wild-type p53, which is responsive to MDM2 inhibition (Fig. 7c, d).

## Discussion

Adrenocortical cancer remains a difficult malignancy to treat. New therapeutic regimens have not been forthcoming in part due to the relative rarity of the disease. Recently, the two major phase III studies, the FIRM-ACT trial and the OSI-906 study have been completed. The FIRM-ACT study was designed to establish a first-line chemotherapy standard which at this point is generally accepted by the ACC community as the combination of EDP-M [15]. The OSI-906 study was done to investigate the efficacy of a small molecule IGF1R inhibitor in patients whose tumors have progressed on multiple drug chemotherapy. Alas, the results did not show improvement in progressive free survival in the treatment arm, although a small number of patients did appear to derive benefit [34]. Clearly, a need still exists for further study and better treatments. The advent of rapid and high throughput methods for genomic analysis offers the potential ability to accelerate the process of identifying therapeutic leads. Candidate agents must still undergo traditional preclinical evaluation and those agents that are promising should then be entered into clinical trials to evaluate their efficacy in ACC.

Our studies on p53 in adrenocortical cancer are, in general, consistent with the findings of others. We found that p53 mutations in adult sporadic ACC are relatively uncommon. Others have previously reported mutation rates of 10–27 % [18–20, 35–37]. The increased incidence of ACC in children who have Li-Fraumeni syndrome, characterized by an inactivating mutation of p53 [38], however, suggests that one should examine the p53 pathway further in adults with ACC. Analysis of our expression array ACC data confirms that there is perturbation of the p53 pathway in many ACC cases, leading to the conclusion that there are mechanisms other than p53 mutation involved [22].

Targeting of other modifiers of p53 activity is a viable approach. One candidate therapeutic compound targeting polo-like kinase 1 (PLK-1) is advanced in this study. Polo-like kinases are a group of highly conserved serine-threonine kinases that regulate cell cycle checkpoints and cell division [39, 40]. PLK-1 is the most extensively studied, and it controls cell passage through M phase of the mitotic cycle [41]. PLK-1 mRNA is over-expressed in most human cancers including those of the breast, lung, stomach, colon and rectum, ovary, pancreas and prostate [42]. Over-expression of PLK-1 in cancers is thought to portend an adverse prognosis [43, 44]. Our analysis of PLK1 expression relative to survival in our expression data [22] combined with that of Giordano et al. [23] confirms this relationship in ACC. In vitro and in vivo experiments have suggested PLK-1 plays a role in carcinogenesis [40, 45]. Inhibition of PLK-1 in vitro leads to cell cycle arrest at G2/M and apoptosis in human cancer cell lines [39, 41]. Several PLK-1 inhibitors are under clinical development including BI-2536, which was investigated in phase II trials [30, 46] with mixed results. Another PLK-1 inhibitor, BI 6727 or volasertib in combination with low-dose cytarabine is being studied in a phase III trial in patients with acute myeloid leukemia, after encouraging results in a phase II trial [47]. Our study offers evidence that BI-2536 may act to decrease levels of mutant p53 while not affecting wild type p53. It has been previously reported that BI-2536 has activity at nanomolar levels (IC_50_ range 0.002–0.025 µM) in cells lines that have either wild type or mutant p53 [30, 33]. It may be that patients with tumors harboring mutant p53 will respond to inhibition of PLK-1 by BI-2536 better than patients whose tumors harbor a wild-type p53. The results from our xenograft model are supportive of this. One attractive hypothesis that warrants investigation is that if one could inhibit mutant p53 and allow cancer cells to undergo apoptosis, one could enhance tumor response to treatment with other chemotherapeutic agents. In this way, a PLK-1 antagonist such as BI-2536 could be used as part of a combination chemotherapeutic strategy to treat ACC.

The mechanism by which BI-2536 affects mutant p53 levels is unclear and should be studied further. Several explanations are possible. PLK-1 may act to stabilize mutant p53 and protect it from degradation or inactivation. Inhibition of PLK-1 may act at the transcription or translation level. Alternatively, BI-2536 may have a direct effect on p53 independent of its effect on PLK-1. Lastly, we cannot assess based on the current experiments whether the effects seen are particular to ACC or whether other tumor types would behave similarly.

We also considered MDM2, a major negative regulator of p53, which was over-expressed at the RNA level in our samples. A single nucleotide polymorphism (SNP) at position 309 with a conversion of T to G generates a strong SP1 binding site and increases MDM2 expression [48, 49]. We did not observe a change in allele frequency from the expected population frequency in our ACC population, nor did we observe a significant increase MDM2 expression in tumors with a G allele compared to tumors homozygous for the T allele. Furthermore, ACC cell lines were relatively insensitive to pharmacological inhibition of MDM2. MDM2, therefore, seems unlikely to be the sole p53 regulator responsible for the disruption of p53 function in ACC. MDM2 remains of interest because agents targeting it, such as nutlins and in particular nutlin-3, are being investigated as anti-cancer drugs. These agents inhibit the interaction of MDM2 and p53 thereby stabilizing p53 leading to cellular senescence [33]. As such nutlin-3 may work best in tumors with a normal or wild-type p53 gene, therefore making it potentially useful in the adult ACC patient population as approximately 75 % of tumors harbor wild-type p53.

We conclude from these preclinical studies that targeting p53 through PLK-1 is an attractive chemotherapy strategy warranting further investigation in adrenocortical cancer.

## Additional file

10.1186/s40169-015-0080-3 Supplemental Methods and Results for studies of TP53 mutation, p53 codon 72 ploymorphism, and MDM2 SNP309 polymorphism in ACC tumors, Supplemental Tables 1–5 and Supplemental Figures 1–5.

## References

[1] Bilimoria KY, Shen WT, Elaraj D, Bentrem DJ, Winchester DJ, Kebebew E (2008). Adrenocortical carcinoma in the United States: treatment utilization and prognostic factors. Cancer.

[2] Fassnacht M, Allolio B (2009). Clinical management of adrenocortical carcinoma. Best Pract Res Clin Endocrinol Metab.

[3] Roman S (2006). Adrenocortical carcinoma. Curr Opin Oncol.

[4] Allolio B, Fassnacht M (2006). Clinical review: adrenocortical carcinoma: clinical update. J Clin Endocrinol Metab.

[5] Fassnacht M, Kroiss M, Allolio B (2013). Update in adrenocortical carcinoma. J Clin Endocrinol Metab.

[6] Fassnacht M, Libe R, Kroiss M, Allolio B (2011). Adrenocortical carcinoma: a clinician’s update. Nat Rev Endocrinol.

[7] Tacon LJ, Prichard RS, Soon PS, Robinson BG, Clifton-Bligh RJ, Sidhu SB (2011). Current and emerging therapies for advanced adrenocortical carcinoma. Oncologist.

[8] Zini L, Porpiglia F, Fassnacht M (2011). Contemporary management of adrenocortical carcinoma. Eur Urol.

[9] Daffara F, De Francia S, Reimondo G, Zaggia B, Aroasio E, Porpiglia F (2008). Prospective evaluation of mitotane toxicity in adrenocortical cancer patients treated adjuvantly. Endocr Relat Cancer.

[10] Netto AD, Wajchenberg BL, Ravaglia C, Pereira VG, Shnaider J, Pupo AA (1963). Treatment of Adrenocortical Cancer with O,P’-Ddd. Ann Intern Med.

[11] Bapat AA, Demeure MJ, Bussey KJ (2012). A fly in the ointment: reassessing mitotane’s role in the treatment of adrenocortical carcinoma. Pharmacogenomics.

[12] Decker RA, Elson P, Hogan TF, Citrin DL, Westring DW, Banerjee TK (1991). Eastern Cooperative Oncology Group study 1879: mitotane and adriamycin in patients with advanced adrenocortical carcinoma. Surgery.

[13] Costa R, Wesolowski R, Raghavan D (2011). Chemotherapy for advanced adrenal cancer: improvement from a molecular approach?. BJU Int.

[14] Berruti A, Terzolo M, Sperone P, Pia A, Della Casa S, Gross DJ (2005). Etoposide, doxorubicin and cisplatin plus mitotane in the treatment of advanced adrenocortical carcinoma: a large prospective phase II trial. Endocr Relat Cancer.

[15] Fassnacht M, Terzolo M, Allolio B, Baudin E, Haak H, Berruti A (2012). Combination chemotherapy in advanced adrenocortical carcinoma. N Engl J Med.

[16] Wandoloski M, Bussey KJ, Demeure MJ (2009). Adrenocortical cancer. Surg Clin North Am.

[17] Hamid T, Kakar SS (2004). PTTG/securin activates expression of p53 and modulates its function. Mol Cancer..

[18] Libe R, Groussin L, Tissier F, Elie C, Rene-Corail F, Fratticci A (2007). Somatic TP53 mutations are relatively rare among adrenocortical cancers with the frequent 17p13 loss of heterozygosity. Clin Cancer Res.

[19] Ohgaki H, Kleihues P, Heitz PU (1993). p53 mutations in sporadic adrenocortical tumors. Int J Cancer.

[20] Waldmann J, Patsalis N, Fendrich V, Langer P, Saeger W, Chaloupka B (2012). Clinical impact of TP53 alterations in adrenocortical carcinomas. Langenbecks Arch Surg..

[21] de Reynies A, Assie G, Rickman DS, Tissier F, Groussin L, Rene-Corail F (2009). Gene expression profiling reveals a new classification of adrenocortical tumors and identifies molecular predictors of malignancy and survival. J Clin Oncol.

[22] Demeure MJ, Coan KE, Grant CS, Komorowski RA, Stephan E, Sinari S (2013). PTTG1 overexpression in adrenocortical cancer is associated with poor survival and represents a potential therapeutic target. Surgery..

[23] Giordano TJ, Kuick R, Else T, Gauger PG, Vinco M, Bauersfeld J (2009). Molecular classification and prognostication of adrenocortical tumors by transcriptome profiling. Clin Cancer Res.

[24] Dias SS, Hogan C, Ochocka AM, Meek DW (2009). Polo-like kinase-1 phosphorylates MDM2 at Ser260 and stimulates MDM2-mediated p53 turnover. FEBS Lett.

[25] Reich M, Liefeld T, Gould J, Lerner J, Tamayo P, Mesirov JP (2006). GenePattern 2.0. Nat Genet.

[26] Irizarry RA, Hobbs B, Collin F, Beazer-Barclay YD, Antonellis KJ, Scherf U (2003). Exploration, normalization, and summaries of high density oligonucleotide array probe level data. Biostatistics..

[27] Johnson WE, Li C, Rabinovic A (2007). Adjusting batch effects in microarray expression data using empirical Bayes methods. Biostatistics..

[28] Harradine KA, Kassner M, Chow D, Aziz M, Von Hoff DD, Baker JB (2011). Functional genomics reveals diverse cellular processes that modulate tumor cell response to oxaliplatin. Mol Cancer Res.

[29] Pfaffl MW (2001). A new mathematical model for relative quantification in real-time RT-PCR. Nucleic Acids Res.

[30] Steegmaier M, Hoffmann M, Baum A, Lenart P, Petronczki M, Krssak M (2007). BI 2536, a potent and selective inhibitor of polo-like kinase 1, inhibits tumor growth in vivo. Curr Biol.

[31] Frost A, Mross K, Steinbild S, Hedbom S, Unger C, Kaiser R (2012). Phase i study of the Plk1 inhibitor BI 2536 administered intravenously on three consecutive days in advanced solid tumours. Curr Oncol.

[32] Ando K, Ozaki T, Yamamoto H, Furuya K, Hosoda M, Hayashi S (2004). Polo-like kinase 1 (Plk1) inhibits p53 function by physical interaction and phosphorylation. J Biol Chem.

[33] Shangary S, Wang S (2009). Small-molecule inhibitors of the MDM2-p53 protein-protein interaction to reactivate p53 function: a novel approach for cancer therapy. Annu Rev Pharmacol Toxicol.

[34] Fassnacht M, Berruti A, Baudin E, Demeure MJ, Gilbert J, Haak H (2015). Linsitinib (OSI-906) versus placebo for patients with locally advanced or metastatic adrenocortical carcinoma: a double-blind, randomised, phase 3 study. Lancet Oncol..

[35] Herrmann LJ, Heinze B, Fassnacht M, Willenberg HS, Quinkler M, Reisch N (2012). TP53 germline mutations in adult patients with adrenocortical carcinoma. J Clin Endocrinol Metab.

[36] Reincke M, Karl M, Travis WH, Mastorakos G, Allolio B, Linehan HM (1994). p53 mutations in human adrenocortical neoplasms: immunohistochemical and molecular studies. J Clin Endocrinol Metab.

[37] Assie G, Letouze E, Fassnacht M, Jouinot A, Luscap W, Barreau O (2014). Integrated genomic characterization of adrenocortical carcinoma. Nat Genet.

[38] Gonzalez KD, Noltner KA, Buzin CH, Gu D, Wen-Fong CY, Nguyen VQ (2009). Beyond Li Fraumeni Syndrome: clinical characteristics of families with p53 germline mutations. J Clin Oncol.

[39] Garland LL, Taylor C, Pilkington DL, Cohen JL, Von Hoff DD (2006). A phase I pharmacokinetic study of HMN-214, a novel oral stilbene derivative with polo-like kinase-1-interacting properties, in patients with advanced solid tumors. Clin Cancer Res.

[40] Santamaria A, Neef R, Eberspacher U, Eis K, Husemann M, Mumberg D (2007). Use of the novel Plk1 inhibitor ZK-thiazolidinone to elucidate functions of Plk1 in early and late stages of mitosis. Mol Biol Cell.

[41] Barr FA, Sillje HH, Nigg EA (2004). Polo-like kinases and the orchestration of cell division. Nat Rev Mol Cell Biol.

[42] Schoffski P (2009). Polo-like kinase (PLK) inhibitors in preclinical and early clinical development in oncology. Oncologist..

[43] Eckerdt F, Yuan J, Strebhardt K (2005). Polo-like kinases and oncogenesis. Oncogene.

[44] Weichert W, Ullrich A, Schmidt M, Gekeler V, Noske A, Niesporek S (2006). Expression patterns of polo-like kinase 1 in human gastric cancer. Cancer Sci.

[45] Holtrich U, Wolf G, Brauninger A, Karn T, Bohme B, Rubsamen-Waigmann H (1994). Induction and down-regulation of PLK, a human serine/threonine kinase expressed in proliferating cells and tumors. Proc Natl Acad Sci USA.

[46] Muller-Tidow C, Bug G, Lubbert M, Kramer A, Krauter J, Valent P (2013). A randomized, open-label, phase I/II trial to investigate the maximum tolerated dose of the Polo-like kinase inhibitor BI 2536 in elderly patients with refractory/relapsed acute myeloid leukaemia. Br J Haematol.

[47] Döhner H, Lübbert M, Fiedler W, Fouillard L, Haaland A, Brandwein JM et al. Randomized, phase 2 trial of low-dose cytarabine with or without volasertib in AML patients not suitable for induction therapy. vol 9. 2014 10.1182/blood-2014-03-560557 PMC4148765 25006120

[48] Bond GL, Hu W, Bond EE, Robins H, Lutzker SG, Arva NC (2004). A single nucleotide polymorphism in the MDM2 promoter attenuates the p53 tumor suppressor pathway and accelerates tumor formation in humans. Cell.

[49] Knappskog S, Lonning PE (2011). Effects of the MDM2 promoter SNP285 and SNP309 on Sp1 transcription factor binding and cancer risk. Transcription..

