# Supplementary material for: Targeting polo-like kinase 1, a regulator of p53, in the treatment of adrenocortical carcinoma
Source: Clin Transl Med. 2016 Jan 11;5:1. doi: 10.1186/s40169-015-0080-3 (PMC4709336; doi:10.1186/s40169-015-0080-3)
Supplement: Supplementary file 1 — 10.1186/s40169-015-0080-3 Supplemental Methods and Results for studies of TP53 mutation, p53 codon 72 ploymorphism, and MDM2 SNP309 polymorphism in ACC tumors, Supplemental Tables 1–5 and Supplemental Figures 1–5. [file 40169_2015_80_MOESM1_ESM.pdf]

**SUPPLEMENTAL INFORMATION**

**Studies of p53 mutations, p53 codon 72 polymorphism, and MDM2 SNP309 polymorphism**

## METHODS

### Clinical Samples

Tumor samples used included a total set of 31 adrenocortical cancer specimens from patients with adult sporadic disease. These samples were from 15 men and 16 women with a mean age of 48.9 years (range 23.3-77.7). The average size of the tumors was 10.8 cm (range of 3 to 21.5cm). These samples included flash frozen tumors collected at the Mayo Clinic in Rochester, Minnesota, the University Hospital Essen (Essen, Germany) and the University of Calgary (Alberta, Canada). The study was conducted under Western Institutional Review Board approved protocol (WIRB #20051769) that required study subjects to sign a consent form.

### Genomic Analysis

Genomic DNA came from a total of 31 tumors and 2 cell lines and from both bulk tumor and nuclei flow-sorted on the basis of DNA content [1]. Analysis of p53 was done by direct PCR sequencing of exons 1-11 of the TP53 gene. As blood lymphocytes or adjacent normal adrenal tissue from the same patient was not available, we adopted a strategy to use flow-cytometry based sorting of nuclei to separate aneuploid tumor nuclei from the diploid nuclei of supporting normal tissue [1]. We then used the diploid DNA as substitute for normal genomic DNA for each patient. Loss of heterozygosity of TP53 was determined in the 11 samples with DNA from sorted diploid and aneuploid nuclei through analysis of the VNTR1 pentanucleotide repeat [2] in intron 1 of TP53 and/or through the codon 72 polymorphism.

### Expression Microarrays

We used previously published data sets from Affymetrix U133 Plus 2 chips that included both normal adrenal and ACC samples annotated with survival data [1, 2] to identify the number of ACC samples over-expressing PLK1. The .CEL files from Affymetrix U133 Plus 2 chips were downloaded from GEO

(GSE10297 and GSE19750). The data were filtered to include only normal adrenal samples without evidence of disease in the contralateral gland and ACC tumor sample from patients over 18 years of age. Using the Expression File Creator in GenePattern [3], the data were normalized by gcRMA[4] with quantile normalization and background subtraction. Batch effects were minimized using COMBAT [5] with the non-parametric option, floored at 0.00001, and log2 transformed. Normalized expression data was extracted for the PLK1 (202240_at) and TP53 (201746_at) probe sets. MDM2 is represented by multiple probes, so we extracted probe sets 211832_s_at, 205386_s_at, 229711_s_at, and 217542_at. The geometric mean of these probe sets was computed for each sample. The relative fold change to the geometric mean of the corresponding normal samples for each study was computed.

## RESULTS

### Analysis for p53 mutation

Mutations of the coding regions of TP53 are uncommon in adult sporadic ACC. A total of 31 ACC and two ACC cell lines, SW-13 and H295R were analyzed for TP53 mutations through a combination of methods. We sequenced exons 1-11 of TP53 amplified from DNA extracted from bulk tumor (21 of 31) as well as from the aneuploid nuclei from flow sorted tumors (11 of the 31) [6]. Of the 31 ACC tumors, one failed sequencing. Four mutations were detected in 3 tumors (Supplemental Tables 1 and 2). Of note, none of the mutations detected have been reported in ACC previously. Because mutations in small tumor cell populations can be missed when sequencing from bulk material, we examined the 6 tumors that were sequenced from both bulk material and sorted aneuploid material for evidence of missed mutations. No additional mutations were detected. We detected no mutation of TP53 in H295R. SW-13 carried the previously documented H193Y mutation. These results are in line with the sequencing done by the Sanger Institute as part of the COSMIC Cell Lines Project [7].

The 11 tumors subject to flow-sorting were also examined for loss of heterozygosity (LOH) of p53 using the diploid cell population as the “normal” control. LOH occurred in 3 of 11 (27%) of the ACC tumors studied. No tumor with LOH also harbored a mutation. Although the SNP variant with a proline

allele of codon 72 has been associated with an increased risk of ACC [8], we did not observe any difference in the proline allele frequency from that of the expected population frequency in our data.

**Analysis of regulator of p53 activity**

The low rate of mutations in p53, the lack of overlap of mutations with LOH, and a lack of an increase in the prevalence of a proline allele in codon 72 in our ACC samples suggests that disruption of the p53 pathway may involve regulators of p53. MDM2 is the predominant negative regulator of p53 activity. Previous studies have shown that conversion of T to G at position 309 generates a strong SP1 binding site and increases MDM2 expression [9, 10]. Most our tumors did over-express MDM2 at the RNA level (Supplemental Table 3). Therefore, we genotyped SNP 309 in MDM2 in the ACC tumors. We did not observe a change in allele frequency from the expected population frequency, nor did we observe a significant increase MDM2 expression in tumors with a G allele compared to tumors homozygous for the T allele (Tables 1 and 3).

**Supplemental Table 1: Clinical parameters, p53 status and MDM2 SNP 309 status in ACC tumors**

| Sample* | Gender | Age (years) | Survival (years) | Tumor Size (cm) | Tumor Weight (g) | Tumor Stage | Tumor Grade | p53 status¶ | Amino acid change | Codon 72 analysis | p53 LOH | MDM2 SNP309 |
|---|---|---|---|---|---|---|---|---|---|---|---|---|
| ACC 1 | M | 69.5 | 14.2 | 5 | 32 | NA | 3 | WT | NA | R/R | ND | T/G |
| ACC 2 | M | 66.5 | 2.7 | 7 | 60 | NA | 2 | WT | NA | R/R | ND | T/G |
| ACC 3 | F | 63.8 | 19/7 | 14 | 845 | NA | 4 | NA | NA | ND | ND | ND |
| ACC 5* | F | 23.3 | 3.0 | 19 | 1100 | 2 | 3 | WT | NA | P/R | ND | T/T |
| ACC 7 | M | 38.0 | 11.9 | 17 | 130 | NA | NA | NA | NA | ND | ND | T/G |
| ACC 8* | F | 56.5 | 0.6 | 9 | 190 | 4 | 4 | WT | NA | R/R | ND | T/G |
| ACC 9* | M | 53.2 | 9.0 | 8 | 195 | 3 | 2 | WT | NA | R/R | ND | T/T |
| ACC 10* | M | 67.8 | 1.7 | 7.6 | 150 | 2 | 4 | g.7520186 C>A; g.7518969 delGT | p.A76S; Frame shift | R/R | ND | T/T |
| ACC 11* | F | 54.2 | 18 | 15 | 890 | 2 | 1 | WT | NA | R/R | LOH | T/T |
| ACC 12* | M | 72.1 | 0.4 | 9.5 | 175 | 4 | 4 | WT | NA | R/P | ND | G/G |

| | | | | | | | | | | | | |
|---|---|---|---|---|---|---|---|---|---|---|---|---|
| ACC 13* | M | 46.9 | 0.1 | 12 | 235 | 4 | 4 | g.7518281 C>T | p.C242Y | P/P | ND | T/T |
| ACC 14 | M | 35.7 | 0.5 | 21 | 1160 | NA | 3 | WT | NA | R/R | ND | T/T |
| ACC 15 | M | 64.9 | 0.4 | 9 | 120 | NA | 3 | WT | NA | P/R | ND | T/T |
| ACC 16 | F | 42.1 | 10.9 | 21 | 885 | NA | 4 | NA | NA | ND | NI | T/T |
| ACC 17* | F | 26.7 | 16.6 | 3 | NA | Recurrence | 4 | WT | NA | R/R | ND | T/G |
| ACC 18 | M | 77.7 | 0.2 | 17 | 1345 | NA | 3 | WT | NA | R/R | ND | T/G |
| ACC 19* | M | 48.5 | 3.1 | 9 | 195 | 2 | 3 | WT | NA | R/R | ND | T/T |
| ACC 20 | F | 62.3 | 7.8 | 15 | 1115 | NA | 4 | WT | NA | R/R | NI | T/T |
| ACC 21* | F | 36.9 | 13.8 | 6.5 | NA | Recurrence | 4 | g.7517848 A>G | p.V272A | R/R | ND | T/T |
| ACC 22* | F | 67.3 | 6.4 | 10 | 230 | 2 | 2 | WT | NA | R/P | LOH | T/T |
| ACC 23* | F | 27.7 | 9.8 | 8 | 149 | 2 | 2 | WT | NA | R/P | N | T/G |
| ACC 24 | M | 43 | 5.0 | 21.5 | 2880 | NA | NA | WT | NA | R/R | ND | T/G |

| | | | | | | | | | | | | |
|---|---|---|---|---|---|---|---|---|---|---|---|---|
| ACC 25 | F | 35.2 | 4.2 | 4 | 20.5 | NA | NA | WT | NA | R/P | N | T/G |
| ACC 26* | F | 51.5 | NA | 11.7 | NA | 2 | 3 | WT | NA | R/R | ND | T/G |
| ACC 27* | M | NA | NA | NA | NA | Recurrence | 2 | WT | NA | R/R | ND | T/G |
| ACC 28* | F | 58 | NA | 9 | 153.8 | 1 | 2 | WT | NA | R/P | N | T/T |
| ACC 45 | F | 32 | NA | 11 | 106 | 2 | 1 | NA | NA | ND | N | T/G |
| ACC 47 | M | 43 | NA | 4 | 60 | Recurrence | 1 | NA | NA | ND | N | T/G |
| ACC 51 | M | 40 | NA | 12 | 480 | NA | 2 | NA | NA | ND | N | T/T |
| ACC 84 | F | 27 | NA | 9.5 | 300 | NA | 1 | NA | NA | ND | N | T/T |
| H295R | NA | NA | NA | NA | NA | NA | NA | WT | NA | ND | ND | NA |
| SW-13 | NA | NA | NA | NA | NA | NA | NA | g.7518997 C>T | p.H193Y | P/P | ND | NA |
| MPI3* | F | 37.0 | NA | NA | NA | 3 | 2 | WT | NA | R/P | ND | NA |

*Sample previously reported in Stephan, EA *et al* (Stephan, et al. 2008) and Demeure, MJ *et al*, Surgery (in press) 2013 (Demeure 2013)
¶ Referenced against NC_000017.9 (chr.17, build hg18)
ND – not done, NA – not available, NI – Not Informative, P – Proline, R - Arginine

**Supplemental Table 2: p53 mutations detected in ACC tumors**

| Tumor No. | Exon | Chromosomal location | Base change | Amino acid change | Type of mutation | Consequences of the mutation |
|---|---|---|---|---|---|---|
| ACC 10 | 4 | 7520186 | g.7520186C>A | p.A76S | Missense | Functional transactivation, disrupts splicing |
| ACC 10 | 6 | 7518969 | g.7518969delGT | - | Frame shift leading to nonsense mutation [#] | NA |
| ACC 13 | 7 | 7518281 | g. 7518281C>T | p.C242Y | Missense | Non-functional transactivation, gain of function and dominant negative activity |
| ACC 21 | 8 | 7517848 | g.7517848A>G | p.V272A | Missense | Partial functionality, reduced transactivation with temperature sensitivity, disrupts splicing |
| SW-13 | 6 | 7518997 | g. 7518997C>T | p.H193Y | Missense | Non-functional transactivation, temperature sensitivity |

* GenBank Acc : NC_000017.9 (chr.17, hg18)

[#] never previously reported in an ACC

**Supplemental Table 3: Fold change of PLK-1 relative to normal adrenal for the microarray expression data**

| | Log2 Fold-change from Normal | | |
|---|---|---|---|
| Tumor | PLK1 | TP53 | MDM2 |
| G-NC069 | 0.00 | 0.31 | -0.08 |
| G-NC088 | 0.00 | -1.07 | 0.01 |
| G-NC092 | 0.00 | 0.76 | 0.06 |
| G-ACC001 | 0.00 | 1.62 | 1.33 |
| G-ACC008 | 25.85 | 1.20 | -0.18 |
| G-ACC011 | 23.41 | 2.52 | -0.41 |
| G-ACC013 | 0.00 | 1.88 | 0.06 |
| G-ACC014 | 25.22 | 2.87 | 2.51 |
| G-ACC017 | 24.82 | 0.93 | 1.01 |
| G-ACC018 | 30.41 | 1.89 | 0.91 |
| G-ACC019 | 29.86 | 3.23 | -0.35 |
| G-ACC032 | 0.00 | 2.55 | 0.04 |
| G-ACC034 | 24.82 | 1.85 | 0.12 |
| G-ACC035 | 0.00 | 0.68 | 0.37 |
| G-ACC038 | 27.68 | 2.00 | -0.02 |
| G-ACC039 | 0.00 | 1.10 | 0.71 |
| G-ACC042 | 29.11 | 2.33 | 0.22 |
| G-ACC046 | 23.40 | 1.03 | 0.53 |
| G-ACC047 | 30.74 | -28.73 | -0.51 |

| | Log2 Fold-change from Normal | | |
|---|---|---|---|
| Tumor | PLK1 | TP53 | MDM2 |
| G-ACC048 | 25.85 | 3.15 | -0.18 |
| G-ACC051 | 24.98 | 1.87 | -0.25 |
| G-ACC053 | 0.00 | 1.91 | 0.28 |
| G-ACC054 | 0.00 | 2.15 | -0.10 |
| G-ACC055 | 25.25 | 1.86 | 0.09 |
| G-ACC056 | 26.33 | 1.87 | 0.93 |
| G-ACC057 | 25.73 | 1.56 | 1.04 |
| G-ACC058 | 28.77 | 2.54 | 0.23 |
| G-ACC084 | 0.00 | 2.25 | 0.08 |
| G-ACC089 | 29.12 | 0.39 | -0.03 |
| G-ACC094 | 29.46 | 1.88 | 2.41 |
| G-ACC098 | 0.00 | 1.62 | 1.58 |
| G-ACC101 | 25.44 | 1.75 | 0.71 |
| G-ACC102 | 26.28 | 1.20 | 0.21 |
| G-ACC103 | 0.00 | 2.21 | 0.17 |
| B-ACC-0520 | -0.01 | 0.07 | 0.18 |
| B-ACC-0523 | 0.02 | -0.16 | 0.00 |
| B-ACC-Norm1 | 0.03 | -0.02 | -0.12 |
| B-ACC-Norm2 | -0.03 | 0.11 | -0.06 |
| B-ACC-5 | 0.13 | 0.18 | 0.32 |

| | Log2 Fold-change from Normal | | |
|---|---|---|---|
| Tumor | PLK1 | TP53 | MDM2 |
| B-ACC-8 | 4.35 | 0.97 | 0.09 |
| B-ACC-10 | 4.00 | -1.29 | 0.39 |
| B-ACC-12 | 0.28 | 0.89 | 1.28 |
| B-ACC-13 | 0.02 | 2.40 | -0.07 |
| B-ACC-17 | 0.03 | 0.56 | 0.18 |
| B-ACC-19 | 0.01 | 1.41 | 2.60 |
| B-ACC-21 | 0.03 | 1.13 | 1.57 |
| B-ACC-26 | 2.48 | 0.38 | 0.34 |
| B-ACC-9 | 0.02 | 0.88 | 0.31 |
| B-ACC-MPI3 | 0.02 | -0.59 | 0.12 |
| B-ACC-11 | 0.00 | 0.75 | 0.30 |
| B-ACC-22 | 0.05 | 0.52 | 0.99 |
| B-ACC-23 | -0.01 | 2.23 | 0.26 |
| B-ACC-27 | -0.01 | 0.76 | 0.98 |
| B-ACC-28 | 0.59 | 1.93 | 0.91 |
| B-ACC-4 | -0.01 | 1.21 | 1.33 |
| B-ACC-MPI1 | 0.00 | 0.74 | 0.02 |
| B-ACC-MPI2 | -0.01 | 1.84 | -0.15 |
| B1-ACC-30 | 0.59 | 1.01 | 1.50 |
| B1-ACC-31 | 0.19 | 0.91 | -0.48 |

| | Log2 Fold-change from Normal | | |
|---|---|---|---|
| Tumor | PLK1 | TP53 | MDM2 |
| B1-ACC-32 | 1.47 | 0.51 | 0.45 |
| B1-ACC-35 | 0.30 | 1.23 | 1.11 |
| B1-ACC-45 | 0.26 | 0.29 | 0.68 |
| B1-ACC-47 | 0.32 | 1.69 | -0.27 |
| B1-ACC-48 | 0.87 | -0.46 | -1.37 |
| B1-ACC-51 | 0.10 | 0.65 | 1.44 |
| B1-ACC-59 | 0.60 | 1.06 | 1.05 |
| B1-ACC-61 | 3.04 | 1.48 | 0.33 |
| B1-ACC-84 | 0.34 | 2.06 | 1.30 |
| B1-ACC-85 | 0.59 | 1.09 | 2.47 |
| B1-ACC-112 | 0.22 | -0.08 | -7.88 |
| B1-ACC-114 | 0.59 | 0.57 | 1.05 |
| B1-ACC-115 | 0.04 | 1.99 | 1.60 |
| B1-ACC-117 | 0.02 | 2.27 | 1.73 |
| B1-ACC-118 | -0.06 | 0.38 | -1.29 |
| B1-ACC-123 | 0.11 | 0.84 | -0.80 |
| B1-ACC-129 | 0.01 | 1.94 | 1.68 |
| B1-ACC-132 | 0.18 | 0.77 | 1.31 |
| B1-ACC-134 | 0.17 | 0.38 | -8.45 |
| B1-ACC-136 | -0.05 | 1.59 | 1.98 |

| | Log2 Fold-change from Normal | | |
|---|---|---|---|
| Tumor | PLK1 | TP53 | MDM2 |
| B1-ACC-140 | 4.72 | -0.11 | -7.59 |
| B1-ACC-149 | 0.07 | 0.30 | -0.48 |
| B1-ACC-150 | 2.34 | 0.17 | -0.39 |

**Supplemental Table 4: Fold-Change Relative to DMSO (vehicle) Control of RT-qPCR Results for TP53 and CDKN1A Expression After PLK1 Inhibition with BI-2536 in H295R**

| | TP53 | | | CDKN1A (p21) | | |
|---|---|---|---|---|---|---|
| Cells Only | 0.0126 | 0.155 | | 0.647 | 3.25 | |
| BI-2536 IC10 | 0.388 | 0.0821 | 0.897 | 0.806 | 0.756 | 0.933 |
| BI-2536 IC25 | 0.472 | 0.0999 | 0.614 | 1.056 | 1.12 | 1.14 |
| BI-2536 IC50 | 0.758 | 0.146 | 1.11 | 1.21 | 1.467 | 1.53 |

**Supplemental Table 5: Fold-Change Relative to DMSO (vehicle) Control of RT-qPCR Results for TP53 and CDNK1A Expression After PLK1 Inhibition with BI-2536 in SW-13**

| | TP53 | | | CDKN1A (p21) | | |
|---|---|---|---|---|---|---|
| Cells Only | 1.11 | 1.32 | | 1.84 | 1.15 | |
| BI-2536 IC10 | 1.16 | 1.05 | 1.25 | 0.705 | 0.862 | 1.51 |
| BI-2536 IC25 | 1.35 | 1.02 | 1.01 | 0.835 | 0.498 | 1.30 |
| BI-2536 IC50 | 0.999 | 0.251 | 1.04 | 0.656 | 0.987 | 1.24 |

**Supplemental Figure 1: Distribution of the log of PLK1 versus the log of β-actin mRNA expression of normal adrenal glands, ACC tumor samples, and the ACC cell lines SW-13 and H295 (parent line to H295R).** The ACC cell lines express comparable amounts of PLK1 mRNA as clinical samples.

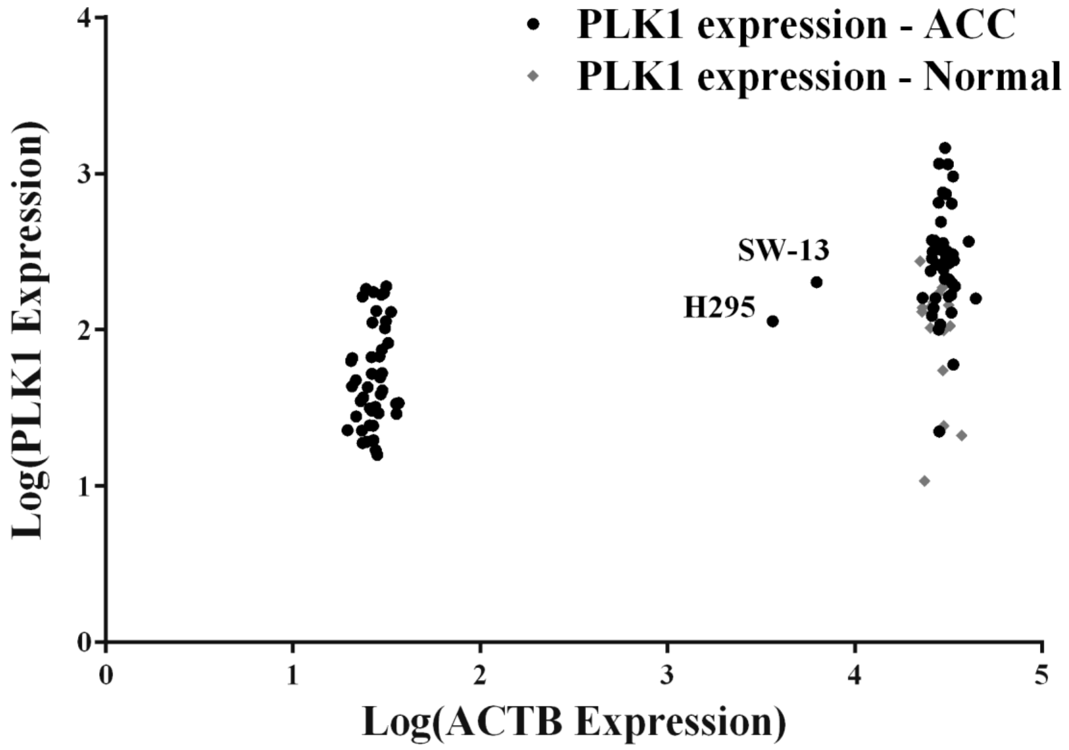

**Supplemental Figure 2: Drug dose response curves for H295R and SW13 in response to MDM2 inhibition with Nutlin-3 at 72, 96, and 120 hour post addition of drug.** Assays were normalized first to cells alone and then to a vehicle control. This resulted in viability measures of greater than 100%.

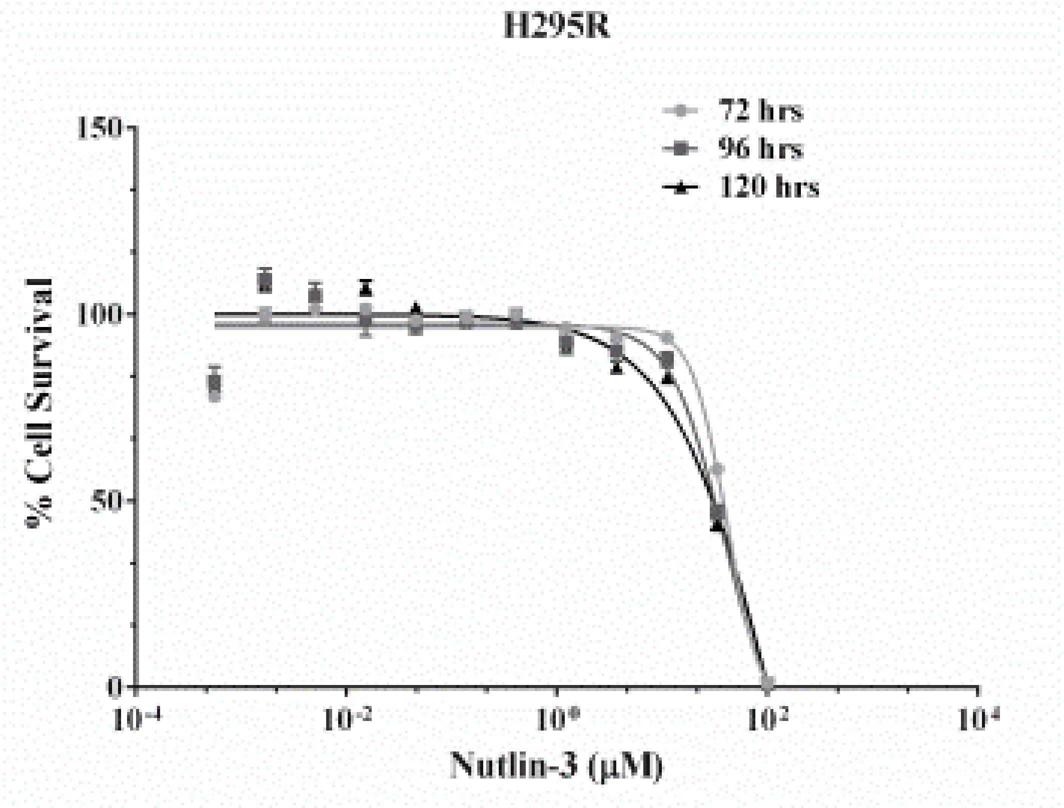


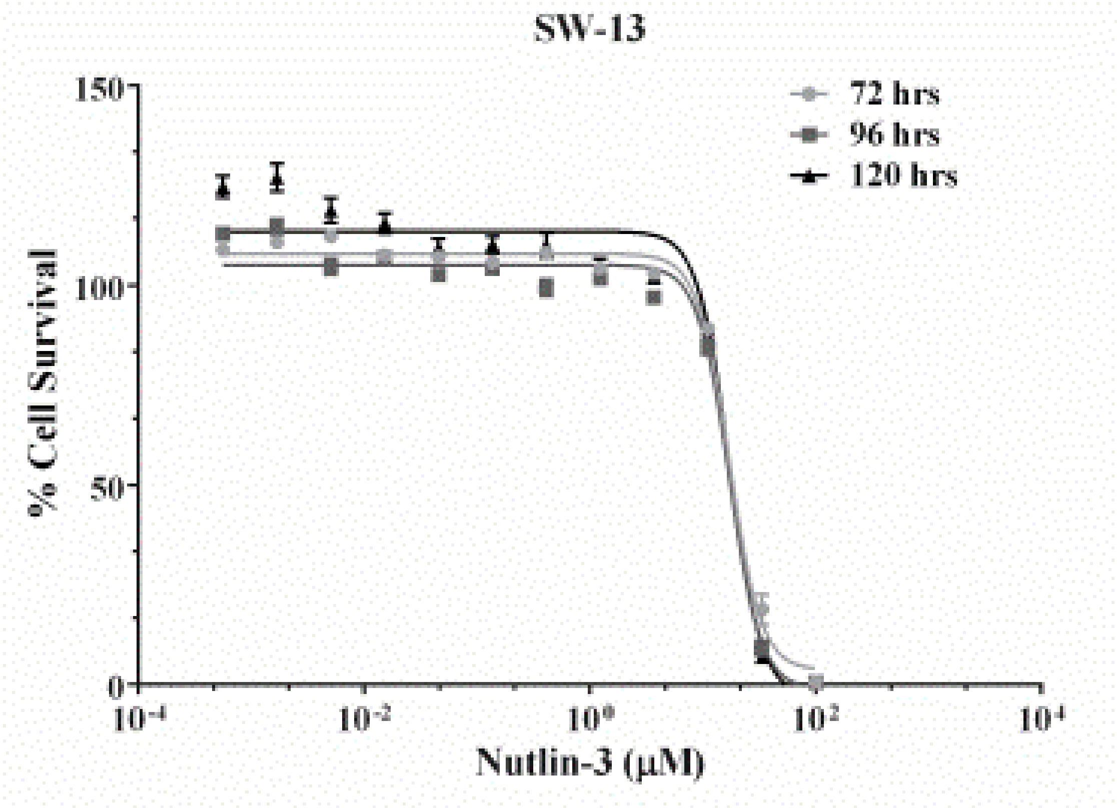

**Supplemental Figure 3:** Western blots for PLK1 & β-Actin after siRNA knockdown in SW13 & H295R. TF - Transfection reagent only; NT- Non-targeting control siRNA; PLK1- PLK1 siRNA.

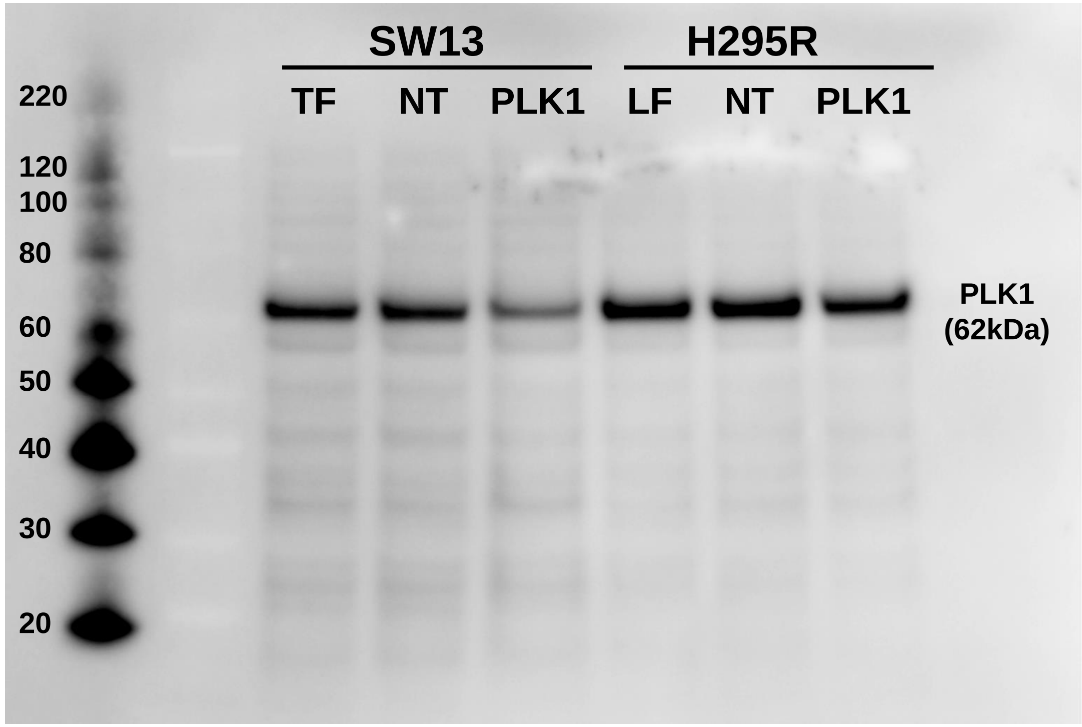


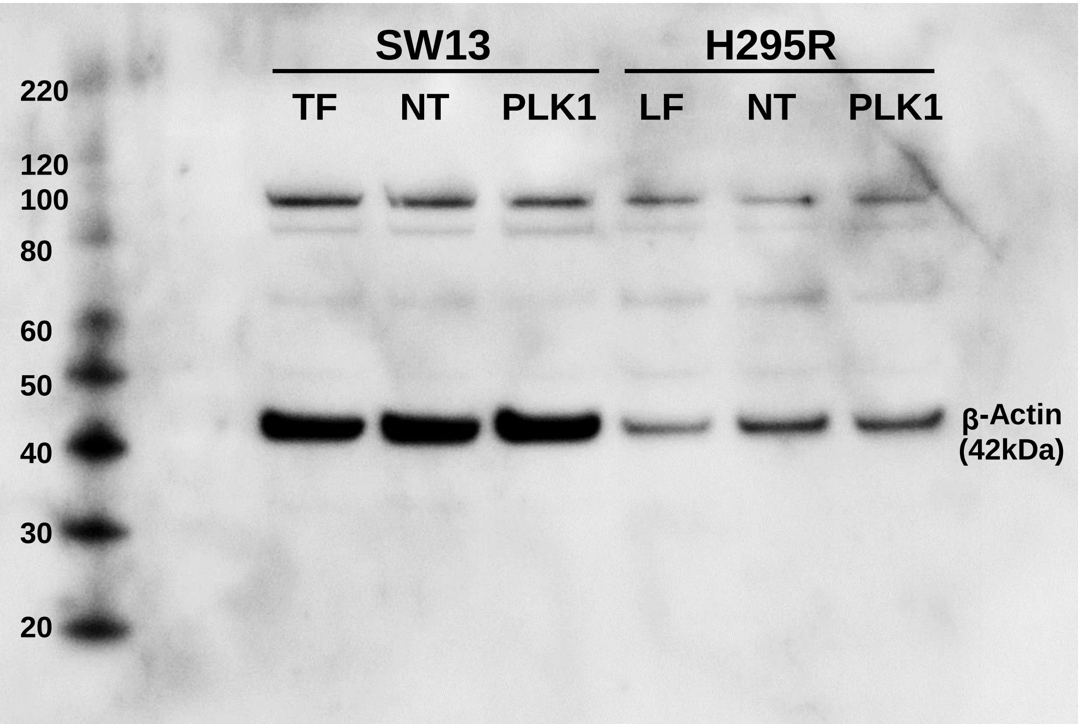

**Supplemental Figure 4:** Western blots for PLK1 & p53 after BI-2536 treatment in SW13 & H295R. Cells – Cells alone. DMSO – Vehicle Control; $IC_{10}$ – $IC_{50}$ – concentration of BI-2536 to which the cells were exposed.

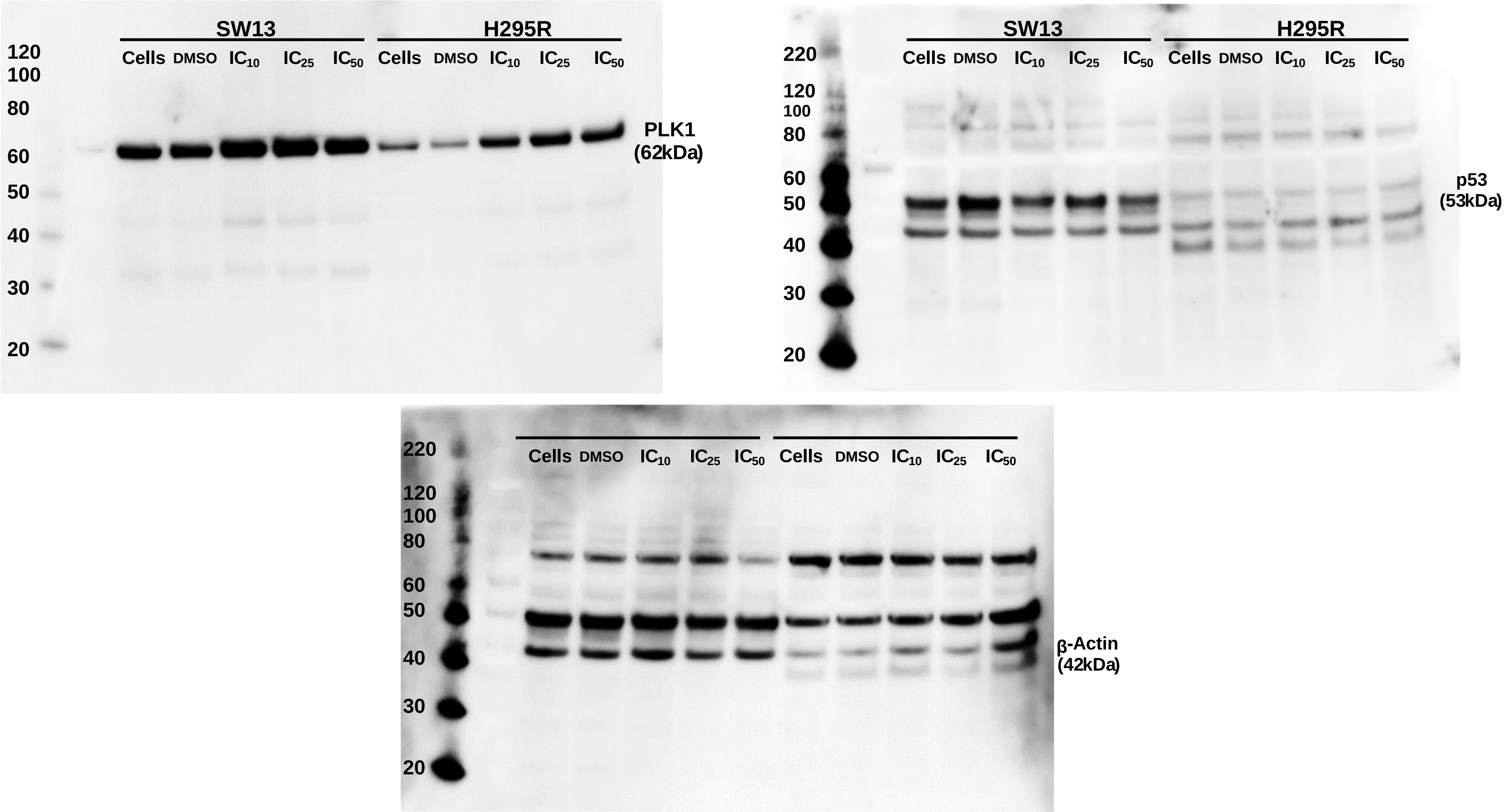

**Supplemental Figure 5:** Full Western blots showing MDM2 proteins levels do not change with increasing concentrations of BI-2536. Cells – Cells alone. DMSO – Vehicle Control; $IC_{10}$ – $IC_{50}$ – concentration of BI-2536 to which the cells were exposed.

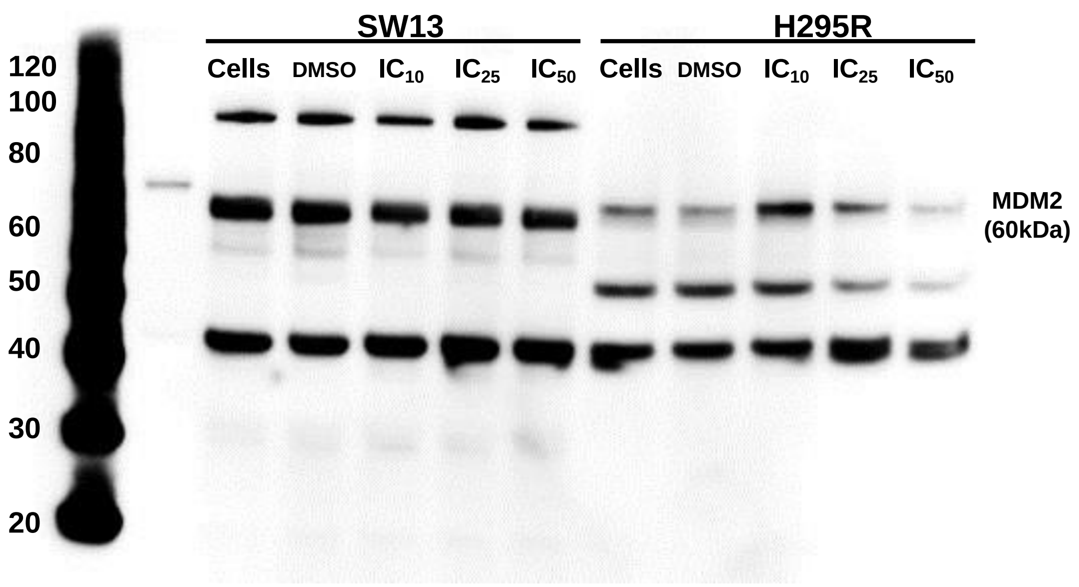


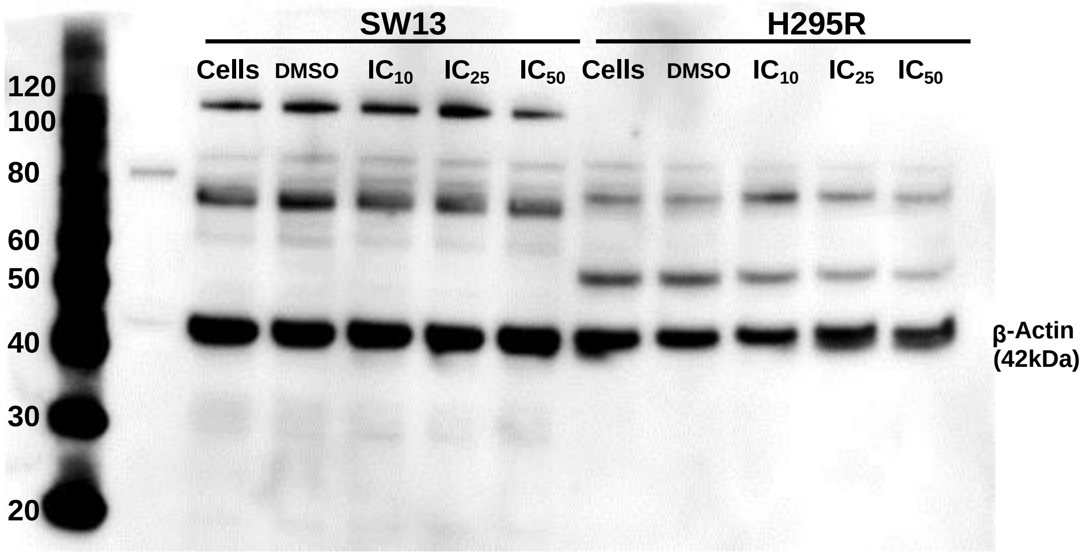

References

1. Demeure MJ, Coan KE, Grant CS, Komorowski RA, Stephan E, Sinari S et al. PTTG1 overexpression in adrenocortical cancer is associated with poor survival and represents a potential therapeutic target. Surgery. 2013;154(6):1405-16; discussion 16. doi:10.1016/j.surg.2013.06.058.

2. Giordano TJ, Kuick R, Else T, Gauger PG, Vinco M, Bauersfeld J et al. Molecular classification and prognostication of adrenocortical tumors by transcriptome profiling. Clin Cancer Res. 2009;15(2):668-76. doi:15/2/668 [pii]
10.1158/1078-0432.CCR-08-1067.

3. Reich M, Liefeld T, Gould J, Lerner J, Tamayo P, Mesirov JP. GenePattern 2.0. Nat Genet. 2006;38(5):500-1. doi:ng0506-500 [pii]
10.1038/ng0506-500.

4. Irizarry RA, Hobbs B, Collin F, Beazer-Barclay YD, Antonellis KJ, Scherf U et al. Exploration, normalization, and summaries of high density oligonucleotide array probe level data. Biostatistics. 2003;4(2):249-64. doi:10.1093/biostatistics/4.2.249
4/2/249 [pii].

5. Johnson WE, Li C, Rabinovic A. Adjusting batch effects in microarray expression data using empirical Bayes methods. Biostatistics. 2007;8(1):118-27. doi:kxj037 [pii]
10.1093/biostatistics/kxj037.

6. Ruiz C, Lenkiewicz E, Evers L, Holley T, Robeson A, Kiefer J et al. Advancing a clinically relevant perspective of the clonal nature of cancer. Proc Natl Acad Sci U S A. 2011;108(29):12054-9. doi:10.1073/pnas.1104009108
1104009108 [pii].

7. Forbes SA, Bhamra G, Bamford S, Dawson E, Kok C, Clements J et al. The Catalogue of Somatic Mutations in Cancer (COSMIC). Curr Protoc Hum Genet. 2008;Chapter 10:Unit 10 1. doi:10.1002/0471142905.hg1011s57.

8. Ignaszak-Szczepaniak M, Horst-Sikorska W, Sawicka J, Kaczmarek M, Slomski R. The TP53 codon 72 polymorphism and predisposition to adrenocortical cancer in Polish patients. Oncol Rep. 2006;16(1):65-71.

9. Bond GL, Hu W, Bond EE, Robins H, Lutzker SG, Arva NC et al. A single nucleotide polymorphism in the MDM2 promoter attenuates the p53 tumor suppressor pathway and

accelerates tumor formation in humans. Cell. 2004;119(5):591-602.

doi:S0092867404010517 [pii]

10.1016/j.cell.2004.11.022.

10. Knappskog S, Lonning PE. Effects of the MDM2 promoter SNP285 and SNP309 on Sp1 transcription factor binding and cancer risk. Transcription. 2011;2(5):207-10.

doi:16813 [pii]

10.4161/trns.2.5.16813.
